# Epidemiological and Clinical Features of Kawasaki Disease During the COVID-19 Pandemic in the United States

**DOI:** 10.1001/jamanetworkopen.2022.17436

**Published:** 2022-06-17

**Authors:** Jennifer A. Burney, Samantha C. Roberts, Laurel L. DeHaan, Chisato Shimizu, Emelia V. Bainto, Jane W. Newburger, Samuel Dominguez, Pei-Ni Jone, Preeti Jaggi, Jacqueline R. Szmuszkovicz, Anne H. Rowley, Nichole Samuy, Paul Scalici, Adriana H. Tremoulet, Daniel R. Cayan, Jane C. Burns, and the KIDCARE Study Investigators

**Affiliations:** 1 School of Global Policy & Strategy, University of California, San Diego, La Jolla; 2 Department of Pediatrics, University of California, San Diego, La Jolla; 3 Rady Children’s Hospital San Diego, La Jolla, California; 4 Scripps Institution of Oceanography, University of California, San Diego, La Jolla; 5 Department of Cardiology, Boston Children’s Hospital, Boston, Massachusetts; 6 Department of Pediatrics, Harvard Medical School, Boston, Massachusetts; 7 Department of Pediatrics, Pediatric Cardiology, Children’s Hospital Colorado, University of Colorado Anschutz Medical Campus, Aurora; 8 Children’s Healthcare of Atlanta, Department of Pediatrics, Emory University, Atlanta, Georgia; 9 Division of Pediatric Cardiology, Children’s Hospital Los Angeles, Keck School of Medicine of the University of Southern California, Los Angeles; 10 Ann & Robert H. Lurie Children’s Hospital of Chicago, Department of Pediatrics, Northwestern University Feinberg School of Medicine, Chicago, Illinois; 11 UAB Heersink, School of Medicine, Department of Pediatrics, University of Alabama at Birmingham, Birmingham

## Abstract

**Importance:**

Public health measures implemented during the COVID-19 pandemic had widespread effects on population behaviors, transmission of infectious diseases, and exposures to environmental pollutants. This provided an opportunity to study how these factors potentially influenced the incidence of Kawasaki disease (KD), a self-limited pediatric vasculitis of unknown etiology.

**Objectives:**

To examine the change in KD incidence across the United States and evaluate whether public health measures affected the prevalence of KD.

**Design, Setting, and Participants:**

This multicenter cohort study included consecutive, unselected patients with KD who were diagnosed between January 1, 2018, and December 31, 2020 (multicenter cohort with 28 pediatric centers), and a detailed analysis of patients with KD who were diagnosed between January 1, 2002, and November 15, 2021 (Rady Children’s Hospital San Diego [RCHSD]).

**Main Outcomes and Measures:**

For the multicenter cohort, the date of fever onset for each patient with KD was collected. For RCHSD, detailed demographic and clinical data as well as publicly available, anonymized mobile phone data and median household income by census block group were collected. The study hypothesis was that public health measures undertaken during the pandemic would reduce exposure to the airborne trigger(s) of KD and that communities with high shelter-in-place compliance would experience the greatest decrease in KD incidence.

**Results:**

A total of 2461 KD cases were included in the multicenter study (2018: 894; 2019: 905; 2020: 646), and 1461 cases (median [IQR] age, 2.8 years [1.4-4.9 years]; 900 [61.6%] males; 220 [15.1%] Asian, 512 [35.0%] Hispanic, and 338 [23.1%] White children) from RCHSD between 2002 and 2021 were also included. The 28.2% decline in KD cases nationally during 2020 (646 cases) compared with 2018 (894 cases) and 2019 (905 cases) was uneven across the United States. For RCHSD, there was a disproportionate decline in KD cases in 2020 to 2021 compared with the mean (SD) number of cases in earlier years for children aged 1 to 5 years (22 vs 44.9 [9.9]; *P* = .02), male children (21 vs 47.6 [10.0]; *P* = .01), and Asian children (4 vs 11.8 [4.4]; *P* = .046). Mobility data did not suggest that shelter-in-place measures were associated with the number of KD cases. Clinical features including strawberry tongue, enlarged cervical lymph node, and subacute periungual desquamation were decreased during 2020 compared with the baseline period (strawberry tongue: 39% vs 63%; *P* = .04; enlarged lymph node: 21% vs 32%; *P* = .09; periungual desquamation: 47% vs 58%; *P* = .16). School closures, masking mandates, decreased ambient pollution, and decreased circulation of respiratory viruses all overlapped to different extents with the period of decreased KD cases. KD in San Diego rebounded in the spring of 2021, coincident with lifting of mask mandates.

**Conclusions and Relevance:**

In this study of epidemiological and clinical features of KD during the COVID-19 pandemic in the United States, KD cases fell and remained low during the period of masking and school closure. Mobility data indicated that differential intensity of sheltering in place was not associated with KD incidence. These findings suggest that social behavior is associated with exposure to the agent(s) that trigger KD and are consistent with a respiratory portal of entry for the agent(s).

## Key Points

**Question:**

How did the incidence and nature of Kawasaki disease (KD) in the United States change during the COVID-19 pandemic?

**Findings:**

In this cohort study of 3922 children with KD, cases of KD across the United States fell by 28% and remained low during periods of COVID-19–related masking and school closure. In the San Diego region, there was a disproportionate decline in KD cases in children aged 1 to 5 years, male children, and Asian children, and clinical features including strawberry tongue, enlarged cervical lymph node, and subacute periungual desquamation were rare.

**Meaning:**

These findings suggest that social behavior is associated with exposure to the agent(s) that trigger KD and are consistent with a respiratory portal of entry for the agent(s).

## Teaser

This cohort study examines the change in Kawasaki disease incidence across the United States and evaluates whether public health measures affected the prevalence of Kawasaki disease.

## Introduction

The COVID-19 pandemic brought Kawasaki disease (KD) into the spotlight in 2 ways, one obvious and one more hidden. The sudden emergence of multisystem inflammatory syndrome in children (MIS-C), a rare but severe condition affecting children 2 to 6 weeks after infection with SARS-CoV-2, was initially confused with KD. Both conditions share an acute innate immune response with high levels of markers of inflammation and shared mucocutaneous signs.^1^ As cases first appeared, KD clinicians were at the forefront of diagnosing MIS-C, delineating its differences from KD, and extrapolating KD knowledge and therapies to this new disease. MIS-C revealed a new illness paradigm, whereby exposure to SARS-CoV-2 was associated with a systemic inflammatory illness several weeks later. The other way in which KD was highlighted by the pandemic was by the reduction in KD case numbers. Recent reports from around the world have revealed significant reductions in KD incidence during 2020.^2,3,4,5,6,7,8^

In this study, we combine analysis of 2 sets of information on KD incidence in the United States before and during the pandemic. In the United States, a multicenter clinical trial for KD (Kawasaki Disease Comparative Effectiveness [KIDCARE] trial; NCT03065244) allowed the prospective collection of date of onset of KD cases across the 28 participating clinical centers from 2018 through 2020.^9^ Additionally, more detailed analyses of population behaviors as well as demographic and clinical details of KD cases were performed at a single pediatric referral center in San Diego using data from time periods before, during, and after the height of the pandemic, from January 2002 to November 2021.

## Methods

### KIDCARE Data Set 

All patients who met the American Heart Association (AHA) definition for complete or incomplete KD from January 1, 2018, through December 31, 2020, were included in this analysis. Date of onset was defined as the first day of fever and was reported monthly for all patients from 28 institutions participating in the KIDCARE trial.^9^ The institutional review board (IRB) at the University of California, San Diego (UCSD; central IRB for trial) and participating sites approved the study. A waiver of informed consent was granted for collection of dates of KD onset because they are not protected health information.

### RCHSD Data Set

Data from the KD Research Center at Rady Children’s Hospital San Diego (RCHSD)/UCSD in San Diego included 1461 patients meeting the strict AHA case definition for complete and incomplete KD, with the exception of 46 individuals (3.3%) from January 1, 2002, to February 28, 2020, and 3 individuals (4.6%) from March 1, 2020, to November 15, 2021. Historically, 95% of all KD cases hospitalized in the San Diego region receive care at RCHSD. One of 2 dedicated KD physicians (J.C.B. and A.H.T.) evaluated each patient during the acute phase and again as outpatients 7 to 21 days after discharge. Prospectively collected data included age at onset, self-reported race and ethnicity (Asian, Black, Hispanic, Indigenous, multiracial, Pacific Islander, unknown, White, and other [including Middle Eastern, North African, and South Asian]), global positioning system coordinates for the patient’s home, and details of the clinical and laboratory evaluation at presentation and over time. The IRB at UCSD approved the study, and parents and participants signed informed consent and assent forms as appropriate. We followed the Strengthening the Reporting of Observational Studies in Epidemiology (STROBE) reporting guideline for cohort studies.^10^

### Demographic and Mobility Data

Analysis of human movement patterns (hereafter, mobility analysis) used information about the timing of public health orders and publicly available, anonymized mobile phone data provided by Safegraph. Safegraph data were used to show the median time spent away from home by residents of all of the census block groups (CBGs) in the 3 counties constituting southern California from January 2019 to April 2021. These data quantified the practice of sheltering in place in different locations and different phases of the COVID-19 pandemic. We used data from the American Community Survey (ACS) on median household income by CBG to relate mobility and KD incidence to neighborhood socioeconomic status. Data were accessed using the TidyCensus package for R version 4.0.4 (R Project for Statistical Computing). For the San Diego time series, we linked Safegraph mobility data by CBG to the CBG where the patient lived at the time of KD onset. This allowed assessment of whether KD cases were from neighborhoods that were sheltering in place more or less intensively.

### Pollution Data

Using Google Earth Engine, we aggregated and downloaded tropospheric column nitrogen dioxide (no_2_) data from the Troposhperic Monitoring Instrument (TROPOMI) on the European Space Agency’s Copernicus Sentinel-5 precursor satellite and found the mean over each CBG at a weekly time scale from January 2019 to October 2021. We merged no_2_ data with the ACS and RCHSD KD data as described previously.

### Circulating Viruses

Data for polymerase chain reaction detection of common viruses by the GenMarkDX ePlex system was provided by the clinical laboratory at RCHSD.

### Statistical Analysis

For all patient counts, we tallied counts by year and compared the 2020 and 2021 values with the distribution of values from the 2002-to-2019 base period (for RCHSD data). For each value, we calculated a *t* statistic as follows: the 2020 or 2021 value minus the mean from 2002 to 2019, divided by the SD from 2002 to 2019. We then calculated the *P* value for this *t* statistic with 18 degrees of freedom. For clinical values, we did not use annual values but rather grouped all patients by time period (ie, 2002-2019, 2020, and 2021) and tested for a difference of medians using Kruskal-Wallis tests. For fractional values (eg, fraction of patients with strawberry tongue), we assessed statistical differences in 2 ways. In some instances, we present the value in 2020 or 2021 with the distribution of annual fractions from 2002 to 2019 for comparison; however, to assess statistical significance, we pooled all values from 2002 to 2019 and used the Fisher exact test to compare proportions from that time period with both 2020 and 2021 (eTable 4 in Supplement 1). For all tests, a 2-tailed *P* ≤ .05 was considered statistically significant.

Although the pandemic-related public health orders did not begin until late February and early March 2020, we analyzed data for 2020 and 2021 as entire years because KD has a strong seasonal component that differs by age group. However, to probe dynamics more fully, we conducted additional exploratory subannual analysis (comparing pandemic-defined periods in 2020 and 2021 with the same seasonal values in the baseline 2002-2019 group). For these analyses, we compared the base period values (mean cases per month ±2 SD) with the 2020 and 2021 values. Because these were small statistics, we report qualitatively when 2020 and/or 2021 values were outside the 95% CI of the baseline period. Statistical differences in mobility and pollution data were also assessed using *t* tests by group and time period.

For Safegraph data, we used *t* tests to evaluate whether there were statistically significant differences in time spent away from home for CBGs that had (or did not have) KD cases in different time periods. A 2-tailed *P*  ≤ .05 was considered statistically significant. Analyses were conducted in R version 4.0.4 (R Project for Statistical Computing).

## Results

### National and Regional KD Incidence

**Figure 1.**
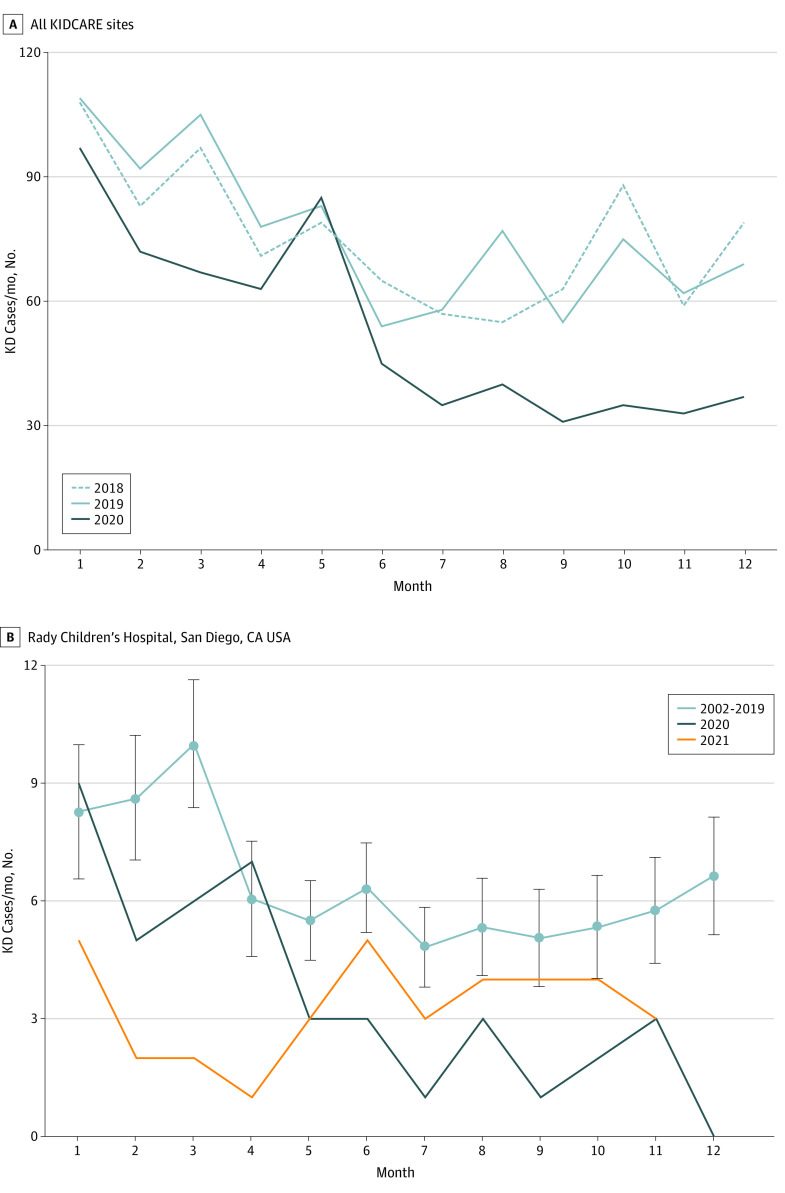
Kawasaki Disease (KD) Incidence Before and During the COVID-19 Pandemic A, Total KD patients for Kawasaki Disease Comparative Effectiveness Trial (KIDCARE) sites: 2018, 894 cases; 2019, 905 cases; 2020, 646 cases. There was a 27.7%-28.6% reduction in case numbers in 2020 compared with previous years. B, Incidence of KD by month of year at Rady Children’s Hospital San Diego during 2002 to 2019. Light blue line is the mean for 2002 to 2019, with errors bars indicating 2 SDs. Cases rebounded to close to within historic levels in late 2021.

Nationally, we studied 2461 patients diagnosed with KD from 2018 to 2020. Across the multicenter study in the United States, the number of KD cases declined in 2020 (Figure 1A), with regional differences in the timing of the reduction in incidence (eTable 1 and eFigure 1 in Supplement 1). Overall, there was a 28.2% reduction in KD cases across these US sites in 2020 (646 cases in 2020 compared with 894 and 905 cases in 2018 and 2019, respectively). This decrease in case numbers was not spatially or temporally homogeneous. Lower case numbers in the winter and spring months of 2020 were driven by western states (Washington and California, excluding San Diego); summertime declines were driven by the Mountain West and Plains, Southeast, and San Diego; and autumn declines were driven by the Midwest and Northeast, with continued lower levels in California and Washington, San Diego, and the Southeast region (eFigure 1 in Supplement 1).

### Demographic and Clinical Features in San Diego

**Figure 2.**
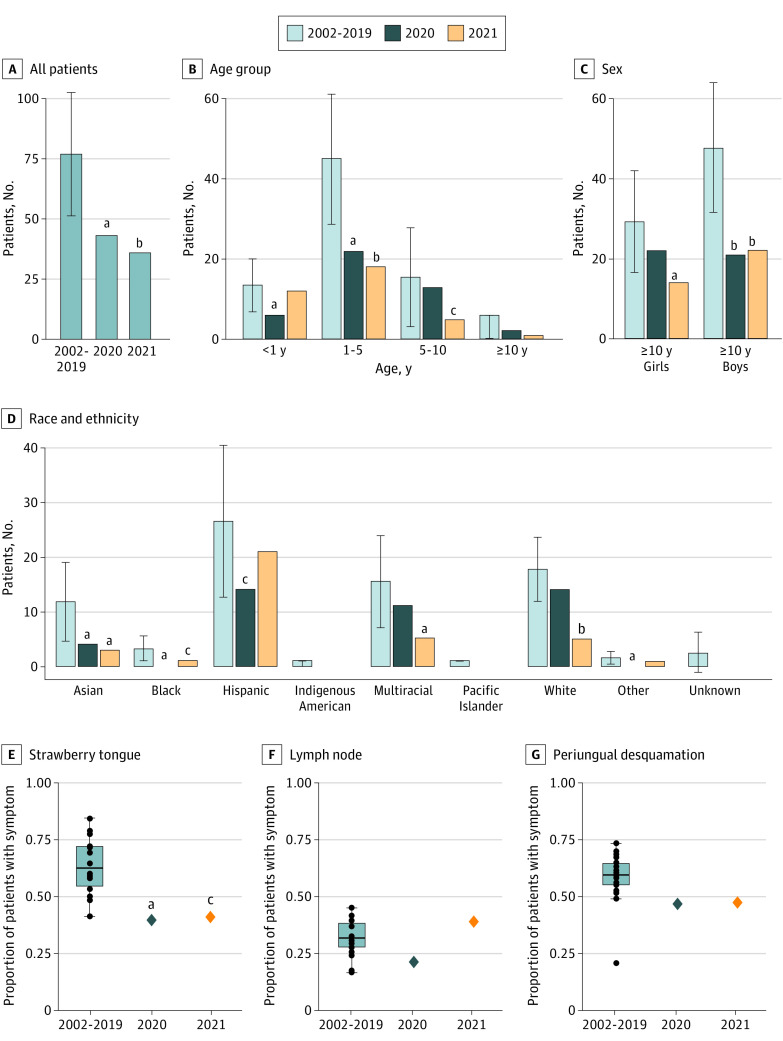
Demographic and Clinical Features of Patients with Kawasaki Disease in San Diego for 2002 to 2019 Compared With 2020 and 2021 A-D, Error bars show the 5th to 95th percentile confidence interval for annual patient counts from 2002 to 2019. B-D, The largest reductions in cases in 2020 were in male children younger than 5 years and among Asian children. In 2021, cases among female and White children also decreased. E-G, Black dots show annual values for 2002 to 2019, with boxes indicating the IQR, the bar indicating the median, and whiskers indicating 1.5 × IQR, for those values. Several clinical features of KD were lower than average in 2020 compared with annual rates in previous years, and strawberry tongue and periungual desquamation remained low through 2021. ^a^*P* < .05. ^b^*P* < .01. ^c^*P* < .10.

We studied 1461 patients (median [IQR] age, 2.8 years [1.4-4.9 years]; 900 [61.6%] males; 220 [15.1%] Asian, 512 [35.0%] Hispanic, and 338 [23.1%] White children) with KD who were diagnosed at RCHSD between January 1, 2002, and December 31, 2021 (Table; eTable 2 in Supplement 1). In the San Diego region, the 2020 to 2021 decline in case numbers was statistically significant compared with the base period (Figure 1B and Figure 2A; eTable 3 in Supplement 1). In 2020, there was a 44% reduction compared with the mean (SD) number of cases in the baseline period (43 vs 76.8 [15.6]; *P* = .02), and for 2021, there was a 53% reduction (36 vs 76.8 [15.6]; *P* = .009). However, this unfolded unevenly for different subsets of patients. The 2020 decline was associated with a reduction in KD compared with the mean (SD) number of cases in the base period for children aged 1 to 5 years (22 vs 44.9 [9.9]; *P* = .02), male children (21 vs 47.6 [10.0]; *P* = .01), and Asian children (4 vs 11.8 [4.4]; *P* = .046). (Figure 2B-D; eTable 4 and eFigures 2-4 in Supplement 1). Although we observed increasing patient age during the 2002-2021 period in the San Diego time series (eFigure 5 in Supplement 1), the older age of patients with KD in 2020 was primarily because of the disproportionate reduction in the number of younger patients with KD (Figure 2B). It should be noted that the timing of the pandemic occurred during the season that could conflate age effects with normal seasonal patterns, which differ slightly by age (eFigure 2 in Supplement 1). Rates of KD for patients younger than 1 year decreased initially but then rebounded (Figure 2B).

**Table.** Characteristics of Kawasaki Disease Cases in Rady Children’s Hospital San Diego Data Set^a^

Characteristic	January 1, 2002, to February 28, 2020 (n = 1396)	March 1, 2020, to November 15, 2021 (n = 65)
Sex, No. (%)		
Male	865 (62)	35 (54)
Female	531 (38)	30 (46)
Age, median (IQR), y	2.8 (1.4 to 4.9)	2.5 (1.0 to 5.3)
Race and ethnicity, No. (%)		
Asian	214 (15)	6 (9)
Black	54 (4)	1 (2)
Hispanic	482 (35)	30 (46)
White	324 (23)	14 (22)
Multiracial	282 (20)	13 (20)
Other^b^	40 (3)	1 (2)
CA worst *z* score, median (IQR)^c^	1.7 (1.1 to 2.5)	1.5 (1.1 to 2.0)
Laboratory data, median (IQR)^d^		
Illness day of lab data collection	6 (4 to 7)	5 (3 to 7)
WBC, /μL	13 200 (10 400 to 17 100)	14 000 (10 300 to 16 200)
ANC, /μL	8580 (6240 to 11660)	9048 (5800 to 11712)
Hemoglobin *z *score^e^	−1.3 (−2.2 to −0.4)	−1.3 (−2.8 to −0.5)
Platelets, ×10^3^/μL	361 (282 to 456)	376 (258 to 506)
ESR, mm/h	60 (39 to 76)	57 (33 to 67)
CRP, mg/dL	6.7 (4.0 to 15.6)	6.0 (4.1 to 16.0)

Abbreviations: ANC, absolute neutrophil count; CA, coronary artery; CRP, C-reactive protein; ESR, erythrocyte sedimentation rate; WBC, white blood cells.

SI conversion factors: To convert ANC and WBC to ×10^9^ cells per liter, multiply by 0.001; CRP to milligrams per liter, multiply by 10; and platelets to ×10^9^ cells per liter, multiply by 1.0.

^a^ *P* values were calculated by *t* test for continuous variables and χ^2^ or Fisher exact *t* test for categorical variables; for sex and race and ethnicity, these tests were conducted on percentages rather than absolute numbers. No significant differences between data sets were found.

^b^ Other included Middle Eastern, North African, and South Asian children.

^c^ The highest *z* score (internal diameter of the right and left anterior descending coronary arteries normalized for body surface area) during the first 6 weeks after fever onset. eTable 2 in Supplement 1 includes information on missing data.

^d^ Laboratory data were measured before treatment. Illness day 1 was defined as the first calendar day of fever.

^e^ The hemoglobin *z *score is the number of SD units from the mean for age-adjusted hemoglobin values.

Clinical subphenotypes of KD in San Diego have been described, and we sought to determine whether there was a shift in the clinical and laboratory patterns of KD during the pandemic compared with previous years^11^ (Figure 2E-G; eFigure 6 in Supplement 1). Although some variability in clinical characteristics was noted across the different time periods, the most notable difference was a reduction in patients with any or all of an enlarged cervical lymph node and strawberry tongue at the time of diagnosis as well as periungual desquamation in the subacute phase during 2020 (Figure 2E-G). Prior to the pandemic, approximately two-thirds of patients with KD had strawberry tongue, and two-thirds had periungual desquamation noted 2 to 3 weeks after fever onset (the notable exception in Figure 2G is the data point from 2019); these features were markedly reduced during the lockdown of 2020 (strawberry tongue: 39% vs 63%; *P* = .04; periungual desquamation: 47% vs 58%; *P* = .16; and enlarged lymph node: 21% vs 32%; *P* = .09) as well as for much of 2021. eFigure 10 in Supplement 1 presents subannual timing. The proportions of all 3 clinical features rebounded in 2021 to greater than prepandemic levels, although the differences were not significant because of the small numbers of patients. There was less variability in laboratory measures of inflammation during the pandemic, with no statistically significant changes observed (eFigure 6 in Supplement 1). Finally, although we observed a small reduction in average coronary artery *z* score for patients through 2021, the fraction of patients with aneurysms did not change.

### Mobility Data for Southern California

**Figure 3.**
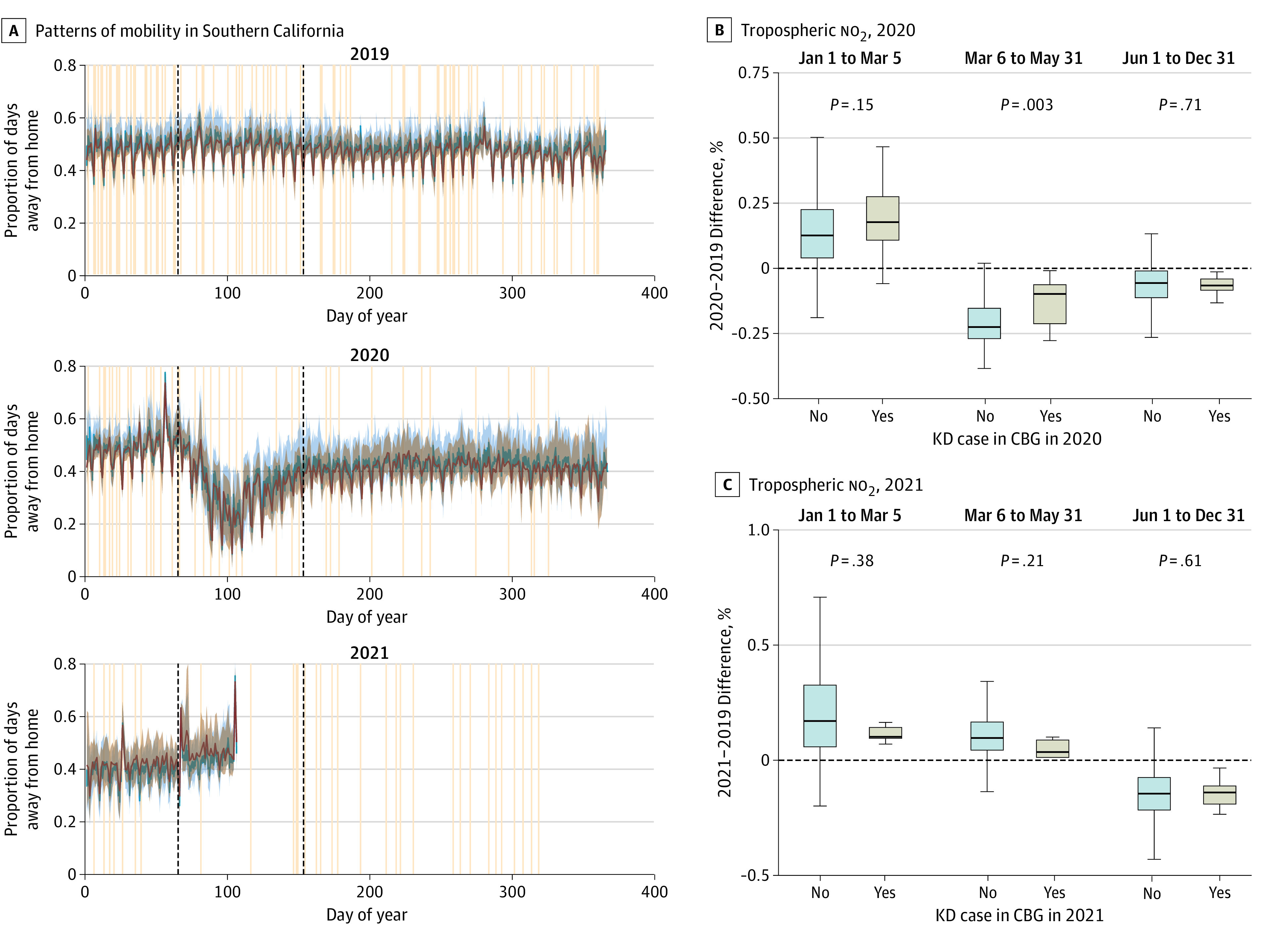
Mobility and Pollution Data A, Patterns of mobility for Southern California were defined as the fraction of the day spent away from home and Kawasaki disease (KD) incidence, 2019 to 2021. Red and blue curves and shading show the median and IQR for the fraction of the day spent away from home for each day in census block groups that had (red) or did not have (blue) KD cases during that year. Tan lines show dates of onset of fever for each KD case at Rady Children’s Hospital San Diego. Black dashed lines show the 2020 pandemic-related shutdown and are included in 2019 and 2021 for comparison. B and C, Changes in tropospheric no_2_ levels relative to the same period in 2019 for CBGs that had or did not have KD cases during that year and pandemic time period. Boxes indicate the IQR; bar, median; and whiskers, 1.5 × IQR. In 2020, during the shutdown period (March 3 to May 31), CBGs with KD cases had significantly smaller reductions in pollution (ie, neighborhoods where the no_2_ levels were more similar to prepandemic levels were more likely to have KD cases during that period).

The pandemic afforded a unique opportunity to examine the combined association of behavioral interventions with the likelihood of exposure to a KD trigger. While compliance with many of these measures was difficult to assess, average neighborhood movement away from home—a proxy for the intensity of sheltering in place—was possible through analysis of cell phone data aggregated by CBG. These data revealed that, on average, across southern California, residents dramatically reduced their movement starting around March 5, 2020 (shutdown vs before shutdown: *P* < .001). Time away from home increased back to steady state but at levels lower than the prepandemic period after approximately June 1, 2020 (eFigure 7 in Supplement 1). Although the differences were small and not statistically significant both before and during the pandemic, the mean time spent away from home for CBGs with KD cases was slightly lower compared with CBGs with no KD cases (Figure 3A; eFigure 8 in Supplement 1).

### Demographic and Clinical Features During Pandemic-Relevant Periods

We used the mobility patterns in 2020 and public health orders to define relevant pandemic periods (Figure 3A). This enabled comparison of 2020 and 2021 to prior years while accounting for both the known seasonality of KD and the very different public health environments over the course of the pandemic. We defined January 1 to March 5 as the prepandemic period in 2020. Although the state of emergency was declared in late February and the statewide shelter-in-place order was issued on March 19, the mobility data showed that behavior changed between those 2 dates as public awareness about COVID-19 grew. We similarly defined March 5 to June 1 as the shutdown period of 2020. This was again informed by the mobility data, as reopening of nonessential businesses began in early June, and the statewide mask mandate was issued June 18, 2020. Vaccinations began for health care workers and older individuals in late December 2020. By April 1, 2021, all adults were eligible for vaccines, and on May 5, 2021, statewide guidance declared that masks were no longer required for fully vaccinated individuals.

Although exploratory because of the small numbers of patients, more detailed analysis of KD incidence (ie, mean number of cases per month) over these pandemic periods revealed intriguing preliminary associations. Overall, KD incidence in San Diego decreased after the initial shutdown and stayed less than the 95th percentile of historical (ie, 2002-2019) levels until summer 2021 (eFigure 2 in Supplement 1). School-aged children (ie, >5 years) followed the same pattern, while younger children (1-5 years) had earlier reductions in incidence during the shutdown period (eFigure 2 in Supplement 1). The fraction of male children with KD also decreased during the initial shutdown period but recovered in spring 2021 (eFigure 3 in Supplement 1).

Patient clinical data at this finer temporal scale revealed no changes in laboratory values by pandemic-defined season (eFigure 9 in Supplement 1). However, the fraction of patients with strawberry tongue fell below the historical 95% CI during the pandemic period in 2020 and stayed outside the historical IQR through 2021. The fraction of patients with enlarged cervical lymph nodes followed a similar, although less extreme, pattern, staying low between the shutdown period in 2020 and spring 2021. The fraction of patients with periungual desquamation in the subacute phase fell during the immediate shutdown period and stayed less than average or outside the historical 95% CI until the second half of 2021 (eFigure 10 in Supplement 1). Although the appearance of these characteristics was reduced, we note that some anomalously low outlier years exist in the historical record (ie, black dots in eFigure 10 in Supplement 1).

For the San Diego region, exploratory analysis also revealed that the geographic location of KD cases shifted during the pandemic compared with previous years, with a concentration of cases along the northern coast of San Diego County (eFigure 11 in Supplement 1). We analyzed the spatiotemporal distribution of KD during the pandemic and its association with socioeconomic status (SES) of the CBG of the patient’s primary residence. In 2019, there was no statistical difference in SES between neighborhoods with and without KD cases. The spatial distribution of KD cases shifted during the pandemic, and KD cases were more likely to occur in neighborhoods with higher SES (median household income of CBGs with vs without KD cases, $90 916 vs $79 250; *P* = .04) (eFigure 8 in Supplement 1). CBG mobility metrics were strongly associated with SES, with residents of wealthier neighborhoods spending more time at home at baseline (slope, −7.6 × 10^−6^; *P* < .001) and reduced time away from home during the initial shelter-in-place period (slope, −1.0 × 10^−6^; *P* < .001). This association was weaker during the rest of the pandemic, with the slope for the second half of 2020 only 29% of the slope during the shutdown period (*P* < .001) (eFigure 12 in Supplement 1).

At an even finer temporal scale, analysis of the day of the week for KD onsets was conducted to determine whether there were hebdomadal patterns, such as a weekend effect, that might suggest exposures associated with triggering the disease. In both the San Diego and national KIDCARE data, there was a small deviation from historical incidence patterns, with one-third of pandemic KD onsets in San Diego occurring on Fridays and higher numbers in both data sets on Wednesdays than in prior years (eFigure 13 in Supplement 1). However, in general, the breakdown of weekday vs weekend onsets remained constant. Day-of-week analysis thus suggested that there was not a weekend effect and complements the lack of a mobility association.

### Environmental Exposures and Circulating Viruses

To understand more about the environmental changes and exposures, we examined both pollution levels and virus circulation preceding and during the pandemic. Previous work has shown that emissions of both greenhouse gasses and air pollutants, particularly from nonstationary sources (transportation) were significantly reduced during the initial shutdown period.^12^ This resulted in lower exposures to both aerosol particulate matter and nitrogen oxides across much of the region during the spring of 2020^13^; it has been further documented that these reductions were greater for lower-income neighborhoods and neighborhoods with higher proportions of Asian and Hispanic residents.^14^ We examined satellite-derived near-surface concentrations of no_2_ (a proxy for all oxides of nitrogen) by CBG over the study period. We found that no_2_ levels for CBGs with KD cases during the shutdown period were reduced relative to the same time in 2019 but to a lesser extent than CBGs without KD cases (Figure 3B). That is, CBGs where pollution levels remained more similar to prepandemic levels were more likely to have cases in the spring of 2020 (reduction in no_2_ levels of CBGs with vs without KD cases, −12.6% vs −21.4%; *P* = .003 during the initial lockdown). Along with mobility, pollution levels rebounded to prepandemic levels by summer 2020, and no statistical difference in change relative to baseline persisted.

**Figure 4.**
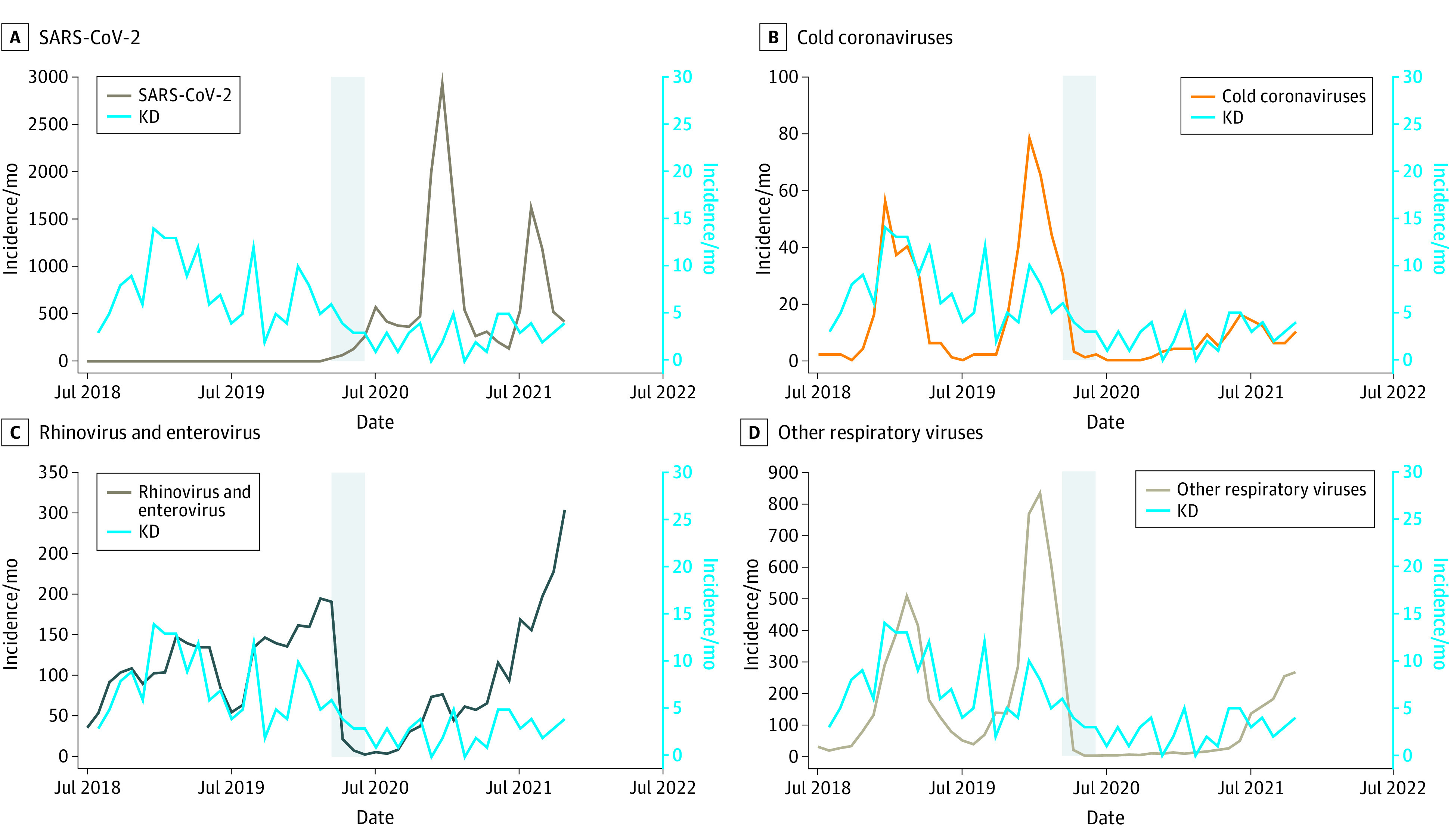
Monthly Incidence of Kawasaki Disease (KD) and Other Viruses Respiratory viruses were detected by polymerase chain reaction in children tested at Rady Children’s Hospital San Diego from July 1, 2018, through November 30, 2021. The initial shelter-in-place period for San Diego County is shown in gray.

In addition to probing how human movement might have changed exposures to the agent(s) that trigger KD, we also conducted an exploratory examination of the prevalence of circulating viruses. Data were available for respiratory virus test results in children seen at RCHSD, a tertiary care facility that serves a population base of approximately 4 million people. The typical seasonal spike in respiratory viruses during the winter months was seen in 2018 to 2019 and 2019 to 2020 (Figure 4). However, respiratory virus infections in children essentially disappeared during the winter of 2020 to 2021. There were no cold coronavirus cases from July through October 2020, and fewer than 1 case per week in November and December as well as April through June. The incidence of other respiratory viruses fell to zero in May and June of 2020, with less than 1 case per week in July and August. The prevalence of rhinovirus/enterovirus infection was less affected compared with other viruses, with a minimum incidence of approximately 1 case per week occurring in June through August of 2020. KD case numbers were reduced but not nearly as much as some respiratory viruses during the shelter-in-place period.

## Discussion

Our goal was to track national temporal trends in KD cases before and during the COVID-19 pandemic and to analyze in depth the association of pandemic-related behavior and environmental changes with KD incidence in the San Diego region. The reduction in KD case numbers coincided with masking, school closures, reduced circulation of respiratory viruses, and reduced air pollution.^13^ A rebound in KD case numbers to prepandemic levels coincided with the lifting of mask mandates and, subsequently, the return to in-person schooling. Although similar reductions in KD cases have been reported in Chicago, Illinois; Asia; and Finland in 2020, the rebound of KD cases in 2021 has not yet been documented from other regions.^2,3,4,6,7,15^ One concern was that the reduction in KD cases could have been related to parental fears about accessing care and the possibility of COVID-19 exposure in health care settings.^16^ However, the report by Ae and colleagues^4^ from Japan showed that the interval between fever onset and diagnosis was unchanged during the pandemic, suggesting that there was no delay in seeking medical attention.

Compared with prepandemic disease patterns in San Diego, the reductions in KD disproportionately occurred in male children, Asian children, and children aged 1 to 5 years. There was no change in the number of infants diagnosed with KD during the pandemic compared with the prepandemic period. This observation was echoed by a report from Japan.^4^ Possible explanations include the fact that infants would not be subjected to pandemic-era behavior changes, such as masking, and changes in exposure to the KD trigger within the home, so decreased mobility would have little effect on KD incidence in this group.

Although our original hypothesis was that shelter-in-place measures would track with reduced KD cases, this was not borne out by the San Diego region data. Instead, the San Diego case occurrence data suggest that exposures that triggered KD were more likely to occur in the home, with a shift toward households with higher SES during the pandemic. The change in mobility by CBG did not account for the decreases in KD case numbers. Thus, the mobility data do not suggest an exposure mechanism associated with an inability to shelter in place as a consequence of employment or socioeconomic pressures. Sheltering in place may have had less impact on KD than other respiratory viruses if the triggering agent(s) are indeed airborne particles. Recent work has documented the relatively weak filtering provided by buildings: high air exchange rates, especially during the day, mean that changes in outdoor aerosol particulate matter are also observed indoors over short time periods.^17^

The reduction in respiratory viruses during the pandemic has been documented from many sites.^2,18,19,20^ The respiratory virus data reported here were uniquely from pediatric patients evaluated in a health care system serving a large catchment area in the San Diego region. Although the majority of respiratory viruses, including the cold coronaviruses essentially disappeared, rhinoviruses/enteroviruses rebounded following the shelter-in-place order, albeit at lower levels compared with prepandemic conditions. Although Hara and colleagues^2^ argued that the disappearance of respiratory viruses argued against their role as a trigger for KD, the rapid rebound of the rhinoviruses/enteroviruses suggests that caution should be exercised in reaching such a conclusion, although no known respiratory virus has been implicated as a KD causative agent. The available data indicates that the trigger(s) for KD enter through the upper respiratory tract.^21^ The role of other aerosols, including pollutants and microbial aerosols, in the etiology of KD remains an open question. Pollution, largely from the transportation sector, decreased during the pandemic in the Southern California region,^13,14^ and the contribution that reactive oxygen species from these pollutants might play in the genesis of KD is unknown, although researchers in Canada have postulated a role.^22^

The observations presented here suggest several productive avenues for research into the etiology of KD. The data suggest that oropharyngeal swabs from patients, particularly infants, coupled with in-home or local air sampling followed by metagenomic sequencing may be instructive. Focusing on the home environment for infants may be more productive, as their exposures are potentially more limited. The pandemic has shown that limiting exposures to aerosols and large droplets through some combination of masking, social distancing, and hand hygiene can reduce the incidence of KD in diverse communities throughout the globe.

### Limitations

This study has limitations. First, small sample sizes limit the strength of conclusions possible from these analyses; this is all the more so in the pandemic as case numbers were reduced. As a result, seasonal breakdowns should be considered exploratory, and mobility and pollution data associated with the pandemic period need to be interpreted with care because of even smaller-than-usual sample sizes. In addition, KD incidence shows high interannual variability (for example, compare Figure 1B, which shows historical mean incidence and confidence intervals for San Diego, with eFigure 1 in Supplement 1, which shows individual year incidence rates for San Diego). This makes statistical determination of anomalous incidence more difficult. Although we were able to capture a national snapshot of KD case numbers before and during the pandemic, we only studied the rebound in case numbers in the San Diego region. Similarly, we did not have access to detailed clinical or mobility data for the national data set.

Mobility data were based on mobile phone data and aggregated to the CBG level (usually a few thousand individuals). Income and pollution data were also aggregated to the CBG level. As such, the extent to which these data reflect the circumstances of individual children (ie, patients with KD) is uncertain. This may be especially the case for cell phone–derived mobility data. Our environmental data were limited to no_2_ exposure, and we did not have detailed aerosol particle data, including specific species. Additionally, it is possible that some of the patients with KD were misclassified as having MIS-C (or vice versa) because of the clinical signs that overlap between the 2 conditions.

## Conclusions

In this study of KD incidence in the United States between 2018 and 2020, the national and local (San Diego region) reduction in KD cases was associated with a period of school closures, masking mandates, decreased ambient pollution, and decreased circulation of respiratory viruses, which all overlapped to different extents with the period of decreased KD cases. KD in San Diego rebounded in the spring of 2021, coincident with the lifting of the mask mandates. The results presented here are consistent with a respiratory portal of entry for the trigger(s) of KD.

## Notes

Supplement 1. **eTable 1.** Participating Sites and Number of KD Cases (2018-2020) Grouped by Region **eTable 2.** Summary of Missing Data From RCHSD Data Set **eTable 3.** RCHSD Patients by Year and Pandemic-Defined Time Period **eTable 4.** Demographic and Clinical Characteristics of Patients With KD at RCHSD, by Time Period **eFigure 1.** KD Across 28 KIDCARE Sites Grouped by Region **eFigure 2.** KD Incidence in 2020-2021 in San Diego (Patients per Month) Compared With 2002-2019 Baseline, Total and by Age Group **eFigure 3.** KD Incidence in 2020-2021 in San Diego (Patients per Month) Compared With 2002-2019 Baseline, Fraction of Male Patients **eFigure 4.** KD Incidence in 2020-2021 in San Diego (Patients per Month) Compared With 2002-2019 Baseline, by Race and Ethnicity **eFigure 5.** Trends in Age of Patients With KD at RCHSD, 2002-2021 **eFigure 6.** Distribution of Laboratory Values for Patients With KD Seen at RCHSD **eFigure 7.** Mobility Patterns Around COVID-19 Pandemic Onset in Southern California **eFigure 8.** KD, Income, and Mobility by Year **eFigure 9.** Laboratory Values for Patients With KD by Pandemic-Defined Season **eFigure 10.** Clinical Presentation of Patients With KD in 2020 and 2021, Compared With 2002-2019 Baseline, by Pandemic-Defined Season **eFigure 11.** Geographic Distribution of Patients With KD in RCHSD Data Set, 2019-2021 **eFigure 12.** Association Between Census Block Group–Level Median Household Income and Average Time Spent Away From Home **eFigure 13.** KD Fever Onset by Day of Week

Supplement 2.
Nonauthor Collaborators
