# Supplementary material for: Epidemiological and Clinical Features of Kawasaki Disease During the COVID-19 Pandemic in the United States
Source: JAMA Netw Open. 2022 Jun 17;5(6):e2217436. doi: 10.1001/jamanetworkopen.2022.17436 (PMC9206189; doi:10.1001/jamanetworkopen.2022.17436)
Supplement: Supplement 1. — eTable 1. Participating Sites and Number of KD Cases (2018-2020) Grouped by Region eTable 2. Summary of Missing Data From RCHSD Data Set eTable 3. RCHSD Patients by Year and Pandemic-Defined Time Period eTable 4. Demographic and Clinical Characteristics of Patients With KD at RCHSD, by Time Period eFigure 1. KD Across 28 KIDCARE Sites Grouped by Region eFigure 2. KD Incidence in 2020-2021 in San Diego (Patients per Month) Compared With 2002-2019 Baseline, Total and by Age Group eFigure 3. KD Incidence in 2020-2021 in San Diego (Patients per Month) Compared With 2002-2019 Baseline, Fraction of Male Patients eFigure 4. KD Incidence in 2020-2021 in San Diego (Patients per Month) Compared With 2002-2019 Baseline, by Race and Ethnicity eFigure 5. Trends in Age of Patients With KD at RCHSD, 2002-2021 eFigure 6. Distribution of Laboratory Values for Patients With KD Seen at RCHSD eFigure 7. Mobility Patterns Around COVID-19 Pandemic Onset in Southern California eFigure 8. KD, Income, and Mobility by Year eFigure 9. Laboratory Values for Patients With KD by Pandemic-Defined Season eFigure 10. Clinical Presentation of Patients With KD in 2020 and 2021, Compared With 2002-2019 Baseline, by Pandemic-Defined Season eFigure 11. Geographic Distribution of Patients With KD in RCHSD Data Set, 2019-2021 eFigure 12. Association Between Census Block Group–Level Median Household Income and Average Time Spent Away From Home eFigure 13. KD Fever Onset by Day of Week [file jamanetwopen-e2217436-s001.pdf]

Supplemental Online Content

Burney JA, Roberts SC, DeHaan LL, et al; KIDCARE Study Investigators. Epidemiological and clinical features of Kawasaki disease during the COVID-19 pandemic in the United States. *JAMA Netw Open.* 2022;5(6):e2217436. doi:10.1001/jamanetworkopen.2022.17436

eTable 1. Participating Sites and Number of KD Cases (2018-2020) Grouped by Region

eTable 2. Summary of Missing Data From RCHSD Data Set

eTable 3. RCHSD Patients by Year and Pandemic-Defined Time Period

eTable 4. Demographic and Clinical Characteristics of Patients With KD at RCHSD, by Time Period

eFigure 1. KD Across 28 KIDCARE Sites Grouped by Region

eFigure 2. KD Incidence in 2020-2021 in San Diego (Patients per Month) Compared With 2002-2019 Baseline, Total and by Age Group

eFigure 3. KD Incidence in 2020-2021 in San Diego (Patients per Month) Compared With 2002-2019 Baseline, Fraction of Male Patients

eFigure 4. KD Incidence in 2020-2021 in San Diego (Patients per Month) Compared With 2002-2019 Baseline, by Race and Ethnicity

eFigure 5. Trends in Age of Patients With KD at RCHSD, 2002-2021

eFigure 6. Distribution of Laboratory Values for Patients With KD Seen at RCHSD

eFigure 7. Mobility Patterns Around COVID-19 Pandemic Onset in Southern California

eFigure 8. KD, Income, and Mobility by Year

eFigure 9. Laboratory Values for Patients With KD by Pandemic-Defined Season

eFigure 10. Clinical Presentation of Patients With KD in 2020 and 2021, Compared With 2002-2019 Baseline, by Pandemic-Defined Season

eFigure 11. Geographic Distribution of Patients With KD in RCHSD Data Set, 2019-2021

eFigure 12. Association Between Census Block Group–Level Median Household Income and Median Time Spent Away From Home

eFigure 13. KD Fever Onset by Day of Week

This supplemental material has been provided by the authors to give readers additional information about their work.

© 2022 Burney JA et al. *JAMA Network Open.*

eTable 1. Participating Sites and Number of KD Cases (2018-2020) Grouped by Region

Participating Sites (n=28)	2018	2019	2020	Total # of KD cases (%)
**West Coast**				
Cedars-Sinai Medical Center, Los Angeles, CA	1	3	1	5 (0.2)
Children's Hospital Los Angeles, CA	44	47	42	133 (5.4)
Children's Hospital Orange County, CA	39	51	26	116 (4.7)
Harbor UCLA, CA	3	2	5	10 (0.4)
Miller Children's Hospital Long Beach, CA	37	38	26	101 (4.1)
Rady Children's Hospital San Diego, CA	74	95	43	212 (8.6)
Seattle Children's, WA	41	36	29	106 (4.3)
Mattel Children’s Hospital, UCLA, CA	8	3	2	13 (0.5)
Benioff Children’s Hospital, UCSF-Oakland, CA	28	27	19	74 (3.0)
Benioff Children’s Hospital, UCSF-San Francisco, CA	8	9	7	24 (1.0)
University of California Davis, CA	13	15	9	37 (1.5)
**Midwest**				
Children's Hospital of Michigan, MI	22	30	26	78 (3.2)
Indiana University,IN	38	30	19	87 (3.5)

© 2022 Burney JA et al. *JAMA Network Open.*

Lurie Children's Hospital, Chicago, IL	52	39	23	114 (4.6)
Nationwide Children's Hospital, Columbus, OH	37	28	30	95 (3.9)
University of Chicago, Chicago, IL	10	5	4	19 (0.8)
**Northeast**				
Boston Children's Hospital, MA	36	43	37	116 (4.7)
Children's National Hospital, Washington DC	60	46	32	138 (5.6)
Maria Fareri Children's, NY	16	12	6	34 (1.4)
**Mountain West & Plains**				
Children's Hospital Colorado, CO	56	63	50	169 (6.9)
University of Nebraska Medical Center, NE	24	20	12	56 (2.3)
University of South Dakota-Sanford, SD	10	2	0	12 (0.5)
University of Utah, UT	30	25	20	75 (3.0)
**Southeast**				
Arkansas Children's Hospital, AR	15	23	5	43 (1.7)
Emory University, Atlanta, GA	137	88	91	316 (12.8)
University of Alabama, AL	39	51	29	119 (4.8)
University of Texas Southwestern, Dallas, TX	7	67	30	104 (4.2)
Vanderbilt, Nashville, TN	19	19	17	55 (2.2)

© 2022 Burney JA et al. *JAMA Network Open.*

**Total**	909	936	646	2461

© 2022 Burney JA et al. *JAMA Network Open.*

eTable 2. Summary of Missing Data From RCHSD Data Set

	1/1/02 - 2/28/20	3/1/20 - 11/15/21
**Characteristic***	**n= 1396**	**n= 65**
Sex	0	0
Age	0	0
Ethnicity	15 (1.1)	0
CA Z-worst	24 (1.7)	0
Strawberry tongue	536 (38.4)	13 (20.0)
Enlarged lymph node	11 (8.0)	0
Peeling	4 (3.0)	1
Illness Day of lab data collection**	118 (8.5)	0
WBC	128 (9.2)	0
ANC	137 (9.8)	0
z-Hgb	131 (9.4)	0
Platelets	129 (9.2)	0
ESR	166 (11.9)	2 (3.1)
CRP	147 (10.5)	1 (1.5)

*: Values are n (%)

**: Laboratory data was only analyzed for patients with laboratory data collected within the first 15 days after fever onset. Patients may arrive at RCHSD after a series of visits to other physicians results in a referral and may thus be first diagnosed after the laboratory data collection time window has passed.

© 2022 Burney JA et al. *JAMA Network Open.*

eTable 3. RCHSD Patients by Year and Pandemic-Defined Time Period

Year	# of pts.	Year	# of pts.				
**2002**	60	**2012**	79				
**2003**	55	**2013**	86				
**2004**	58	**2014**	95				
**2005**	60	**2015**	82				
**2006**	57	**2016**	111				
**2007**	63	**2017**	83	**Pandemic-defined time periods**			
**2008**	77	**2018**	74		**01/01 - 03/05**	**03/06 - 05/31**	**06/01 - 12/31**
**2009**	80	**2019**	95	**2002-2019 (average)**	341 (18.9)	354 (19.7)	687 (38.2)
**2010**	90	**2020**	43	**2020**	16	14	13
**2011**	77	**2021**	36	**2021**	7	6	23
**Total = 1461**				**Total**	**364**	**374**	**723**

eTable 4. Demographic and Clinical Characteristics of Patients With KD at RCHSD, by Time Period

These data are presented graphically in Figure 2 in the main manuscript. For patient counts: for the 2002-2019 base period, average value (% of total) are shown; for 2020 and 2021, raw numbers (% of total) are shown.

	Base Period 2002-2019 (average)	2020	2021	p-value (2020-Base)	p-value (2021-Base)
**Total**	76.8	43	36	**0.022**	**0.009**
**Age Group**					
< 1 year	13.4 (17.4)	6 (14.0)	12 (33.3)	**0.045**	0.370
1 to 5 years	44.9 (58.5)	22 (51.2)	18 (50.0)	**0.016**	**0.007**
5 to 10 years	15.4 (20.0)	13 (30.2)	5 (13.9)	0.377	0.091
At least 10 years	3.1 (4.1)	2 (4.7)	1 (2.8)	0.266	0.121
**Fraction Male**	0.6	0.49	0.61	**0.013**	0.423
**Race/Ethnicity**					
Afr. American	3.2 (4.1)	0 (0)	1 (2.3)	**0.017**	0.066
Asian	11.8 (15.4)	4 (11.1)	3 (7.0)	**0.046**	**0.030**
Hispanic	26.5 (34.5)	14 (38.9)	21 (48.8)	0.079	0.262
Indigenous American	1.0 (1.3)	0 (0)	0	NA	NA
Mixed	15.5 (20.2)	11 (30.6)	5 (11.6)	0.197	**0.028**
Other	1.5 (2.0)	0 (0)	1 (2.3)	**0.019**	0.219
Pacific Islander	1.0 (1.3)	0 (0)	0 (0)	NA	NA
Unknown	2.5 (3.3)	0 (0)	0 (0)	0.141	0.141
White	17.7 (23.1)	14 (38.9)	5 (11.6)	0.154	**0.001**
**Clinical Characteristics**					
Fraction Peeling	0.58	0.47	0.47	0.160	0.224
Fraction w/ Strawberry Tongue	0.63	0.39	0.41	**0.019**	0.074
Fraction w/ Lymph Node	0.32	0.21	0.39	0.135	0.474
**Laboratory Values**					
WBC	13.2	12.5	13.7	0.922	0.565
ANC	8580	9585	8286	0.402	0.587
PLT	362	361	382	0.905	0.835
CRP	6.7	5.75	6.75	0.531	0.646
ESR	60	53	62	0.108	0.530
Z-hemoglobin	-1.33	-1.38	-1.90	0.307	**0.035**

Values for Age Group and Ethnicity are n (%). Values for Fraction Male and Clinical Characteristics are fractions (0 to 1). Laboratory values are medians. Laboratory data was only analyzed for patients with laboratory data collected within the first 15 days after fever onset. Patients may arrive at RCHSD after a series of visits to other physicians results in a referral and may thus be first diagnosed after the laboratory data collection time window has passed. Bold text for p-values indicates p<0.05.

© 2022 Burney JA et al. *JAMA Network Open.*

eFigure 1. KD Across 28 KIDCARE Sites Grouped by Region

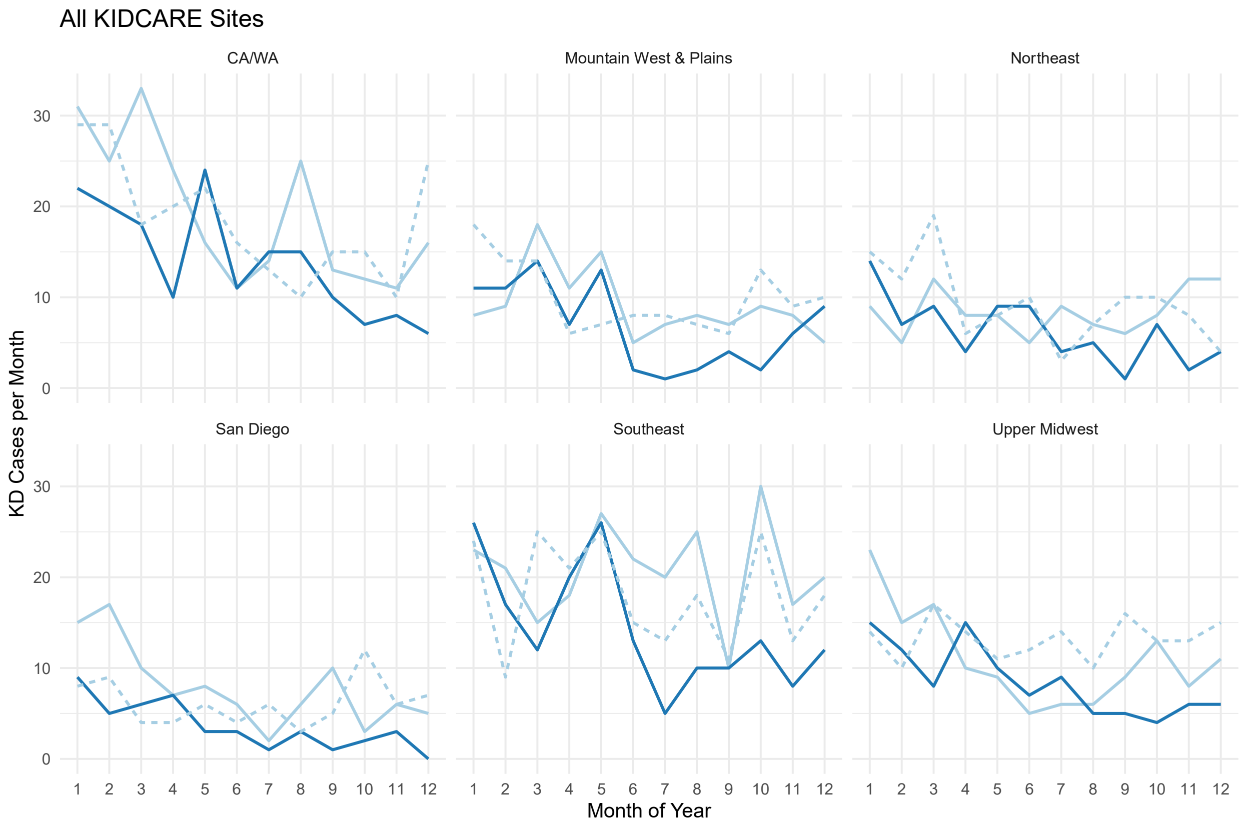

KD incidence: 2018: light blue dashed lines; 2019: light blue solid lines; 2020: dark blue lines. Sites included in each region are listed in Table 1 in the main manuscript.

© 2022 Burney JA et al. *JAMA Network Open.*

eFigure 2. KD Incidence in 2020-2021 in San Diego (Patients per Month) Compared With 2002-2019 Baseline, Total and by Age Group

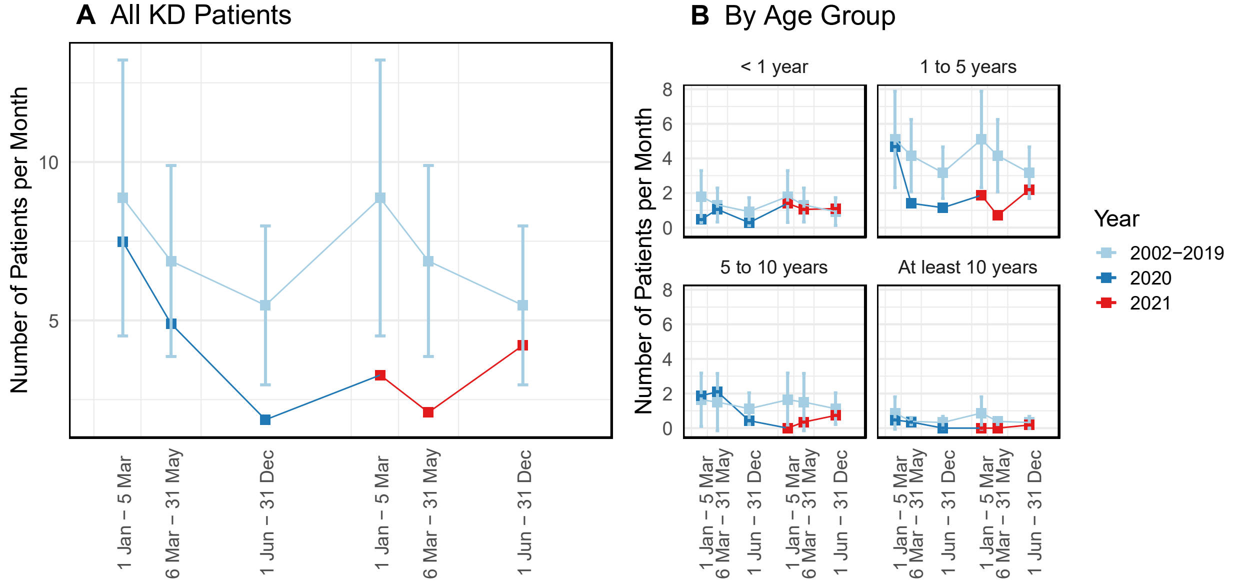

As in the main manuscript, dark blue = 2020 and red = 2021; these are shown in comparison to 2002-2019 historic average +/- 2 SE (light blue). Data are shown over a two-year cycle (the light blue historical values are repeated) to cover the pandemic period. Temporal aggregation is defined by 2020 pandemic-relevant periods. By March 5th, the emergence of a clear decline in human movement around San Diego County marked the beginning of the initial shutdown period, which lasted until early June. In April 2021, all adults became eligible for vaccines, and thereafter CDC issued guidance that vaccinated individuals could stop masking. Dividing the data into these pandemic-defined periods is helpful since KD also has a known seasonality (Figure 1, main manuscript).

© 2022 Burney JA et al. *JAMA Network Open.*

eFigure 3. KD Incidence in 2020-2021 in San Diego (Patients per Month) Compared With 2002-2019 Baseline, Fraction of Male Patients

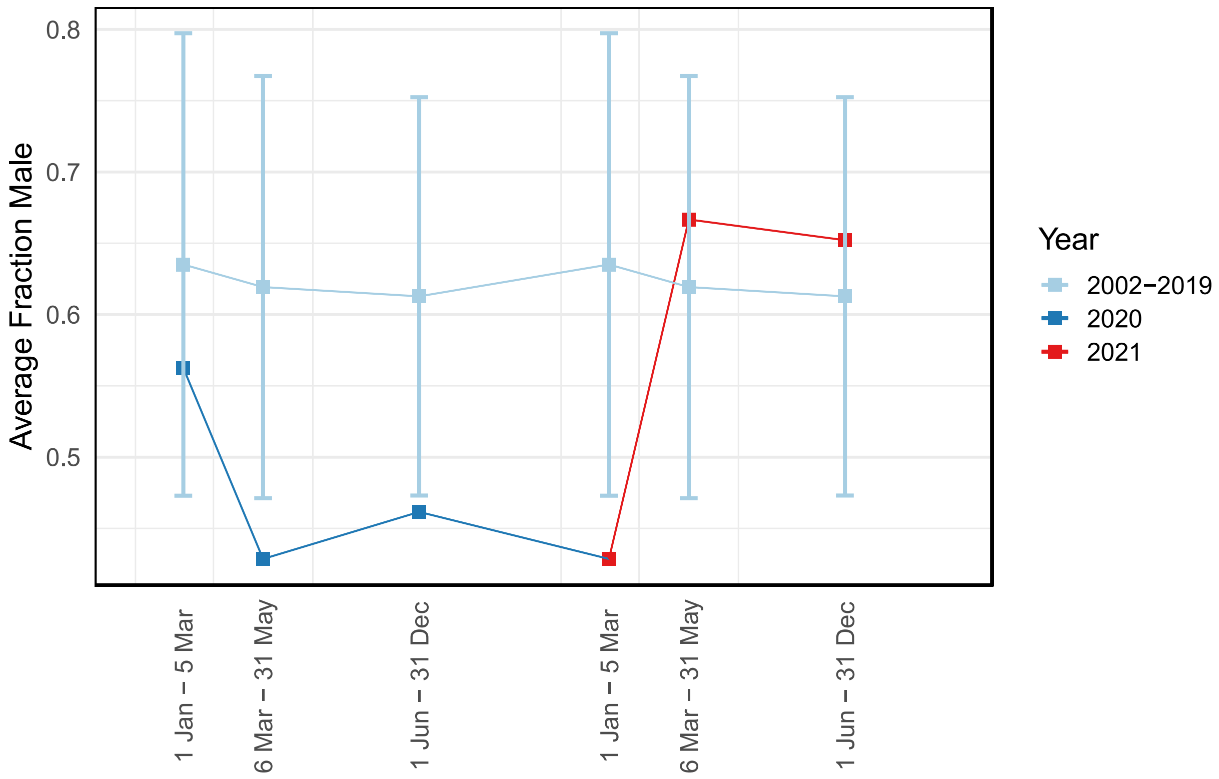

As in Supplemental Figure 2, but for the fraction of KD patients that were male.

© 2022 Burney JA et al. *JAMA Network Open.*

eFigure 4. KD Incidence in 2020-2021 in San Diego (Patients per Month) Compared With 2002-2019 Baseline, by Race and Ethnicity

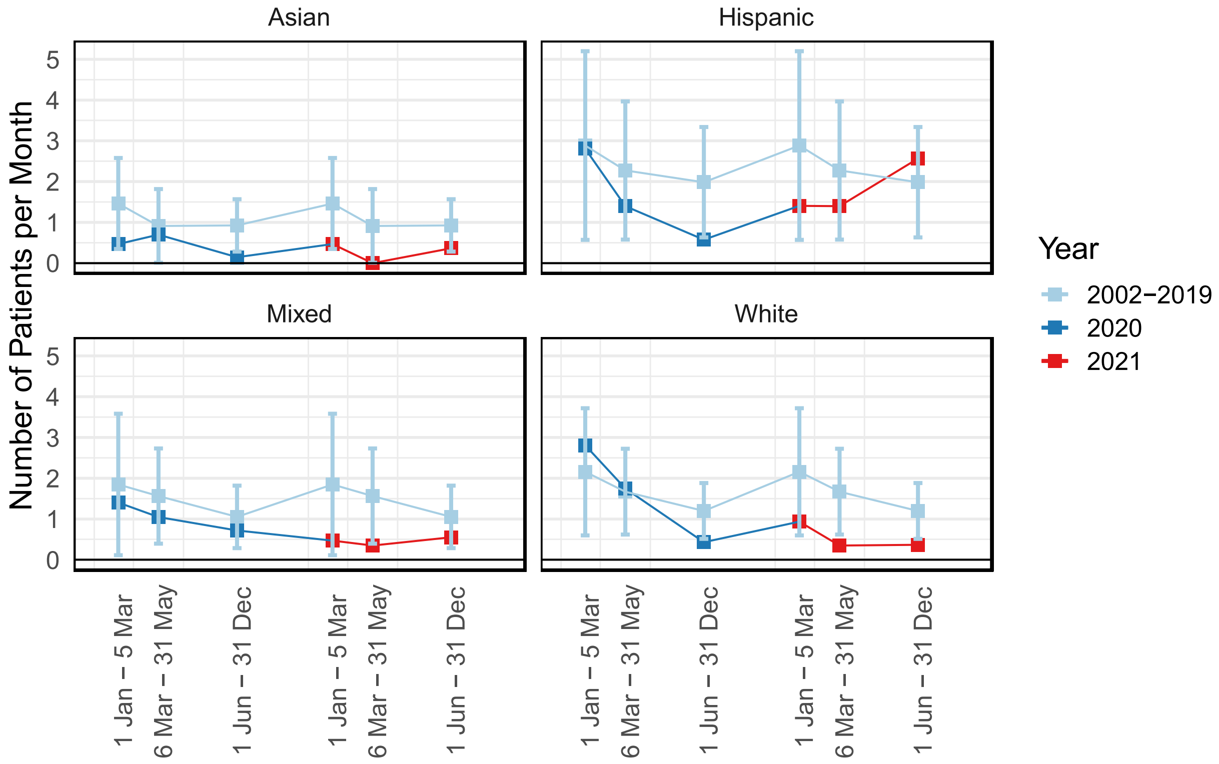

As in Supplemental Figure 2, but for the race/ethnicity of KD patients (groups with highest incidence shown).

© 2022 Burney JA et al. *JAMA Network Open.*

eFigure 5. Trends in Age of Patients With KD at RCHSD, 2002-2021

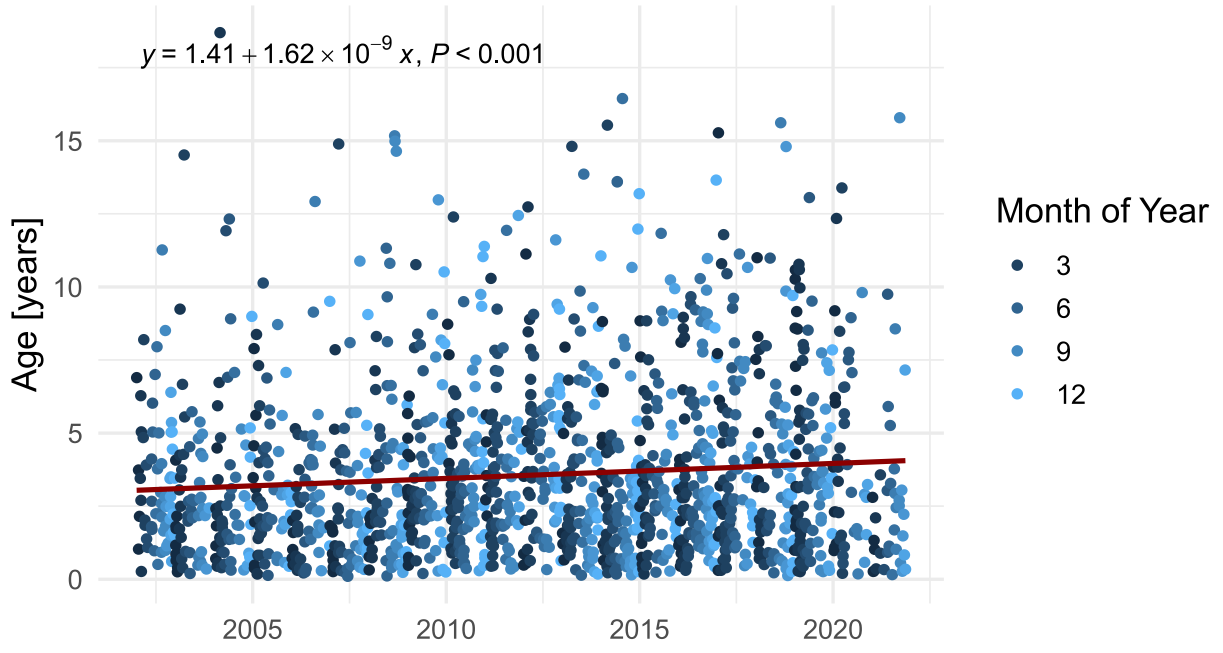

The age of KD patients in San Diego has increased over time. Shade of blue indicates quarter of the year (dark=Q1, lightest=Q4), to aid in qualitatively showing seasonality of incidence.

© 2022 Burney JA et al. *JAMA Network Open.*

eFigure 6. Distribution of Laboratory Values for Patients With KD Seen at RCHSD

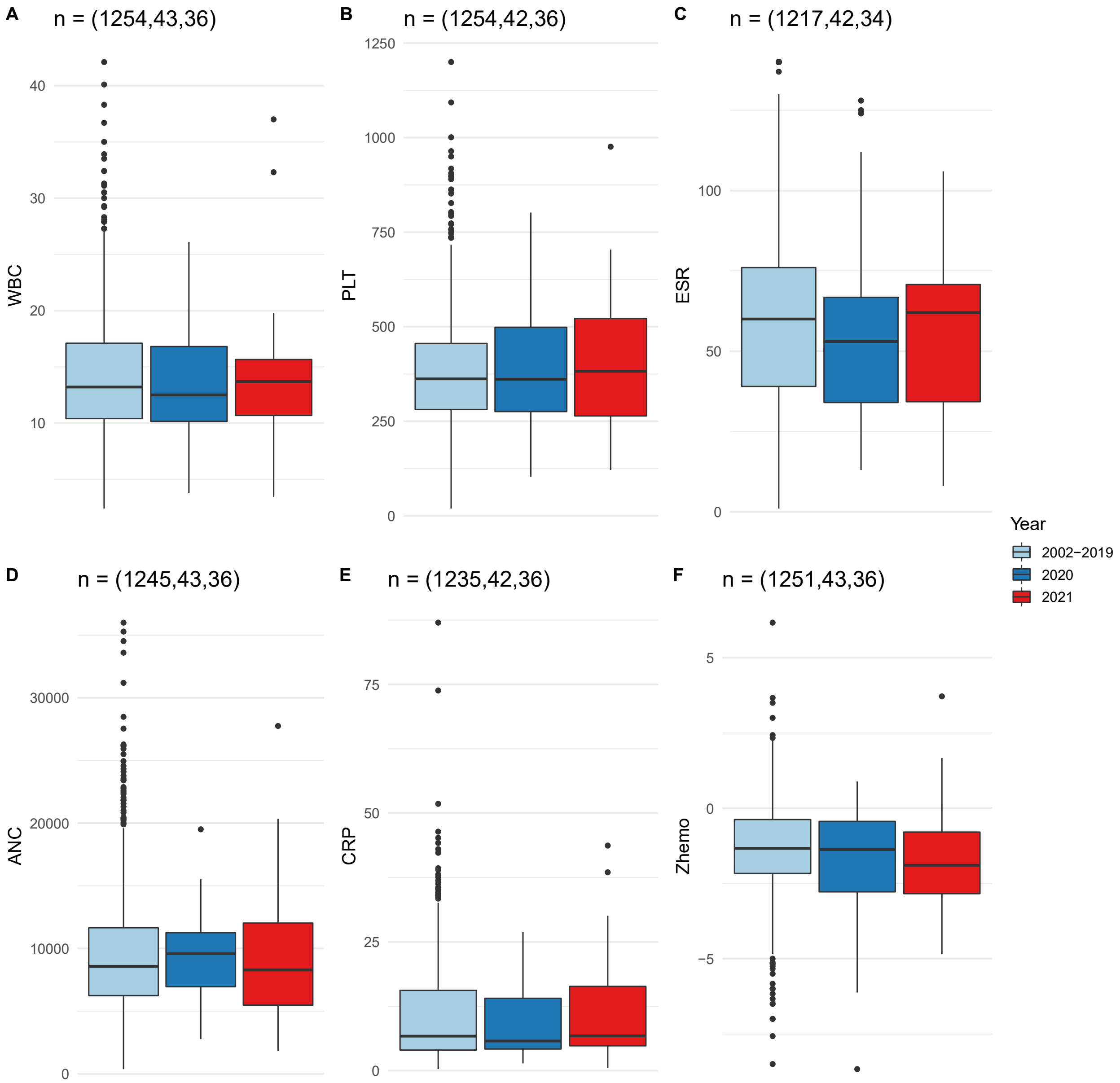

A-F: WBC, Platelets, ESR, ANC, CRP, and Hemoglobin Z-score (hemoglobin concentration normalized for age), respectively, shown for the historical period (2002-2019), 2020, and 2021. Boxes show the IQR for each value, and whiskers show the 95th percentile confidence interval; values beyond that are shown as dots. Numbers of patients in each time period (2002-2019, 2020, 2021) are provided above each panel; full description of missing data is provided in eTable 2. Abbreviations: WBC: white blood cell count, ESR: erythrocyte sedimentation rate, ANC: absolute neutrophil count, CRP: C-reactive protein, Zhemo: hemoglobin Z-score.

© 2022 Burney JA et al. *JAMA Network Open.*

eFigure 7. Mobility Patterns Around COVID-19 Pandemic Onset in Southern California

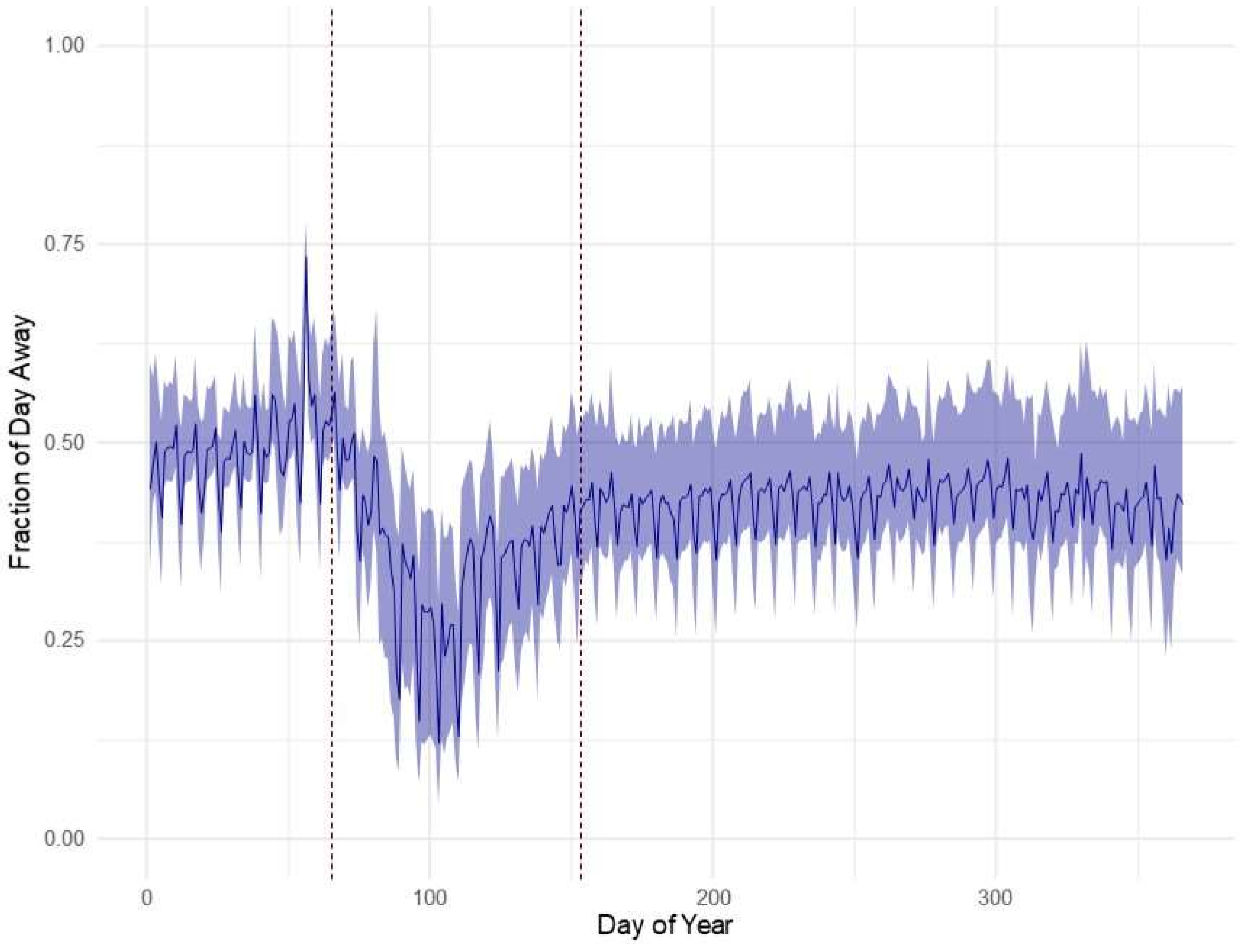

Mobility, defined as the fraction of the day spent away from home, for all southern California Census Block Groups by day for 2020. Dark line shows the median value, with the shaded range showing the IQR.

© 2022 Burney JA et al. *JAMA Network Open.*

eFigure 8. KD, Income, and Mobility by Year

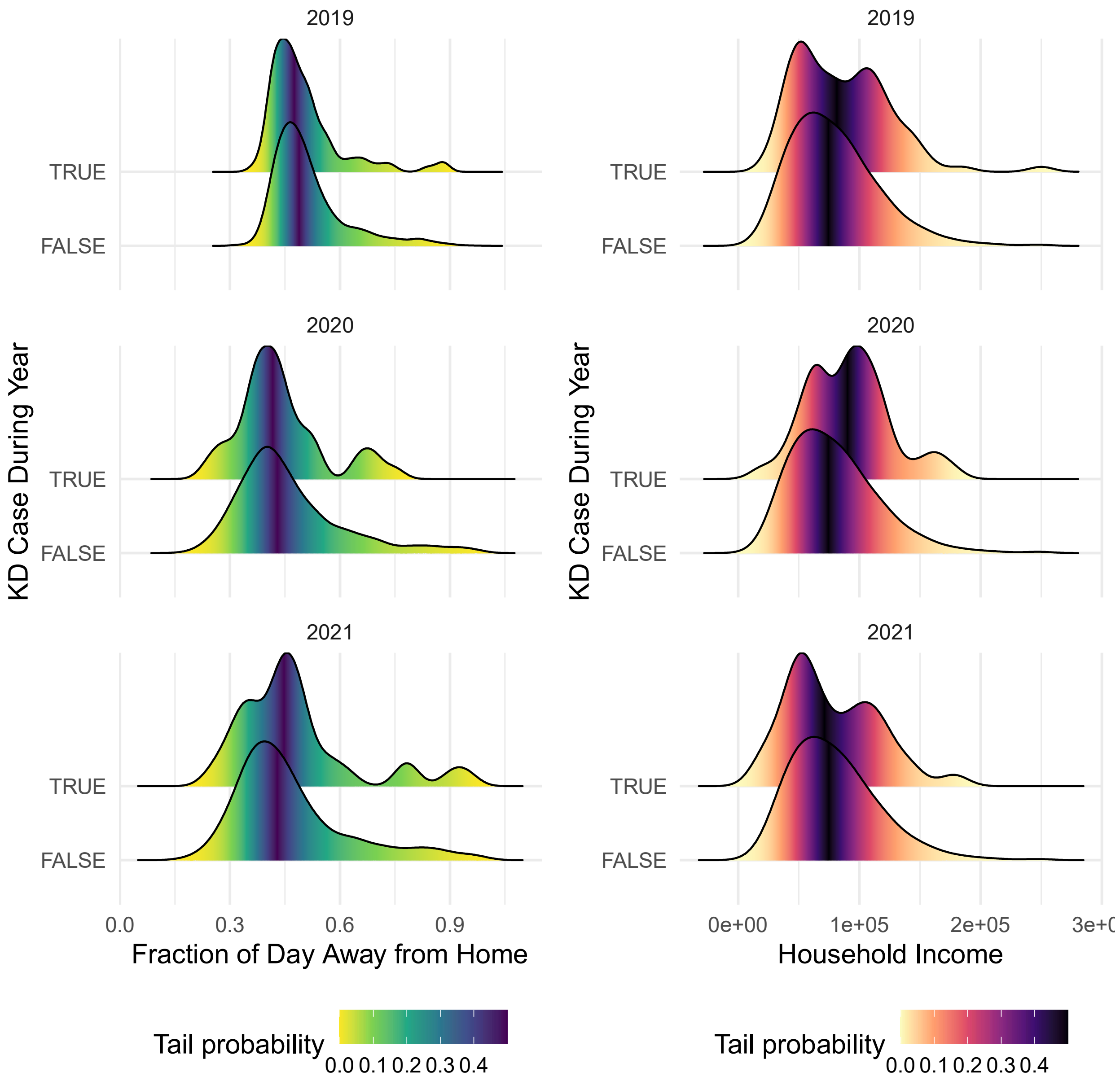

Mobility and income distributions for CBGs that had (TRUE) or did not have (FALSE) KD cases during each year. Mobility differences are not statistically significant (p>0.1); the difference in median CBG household income in 2020 is statistically significant (p=0.037).

© 2022 Burney JA et al. *JAMA Network Open.*

eFigure 9. Laboratory Values for Patients With KD by Pandemic-Defined Season

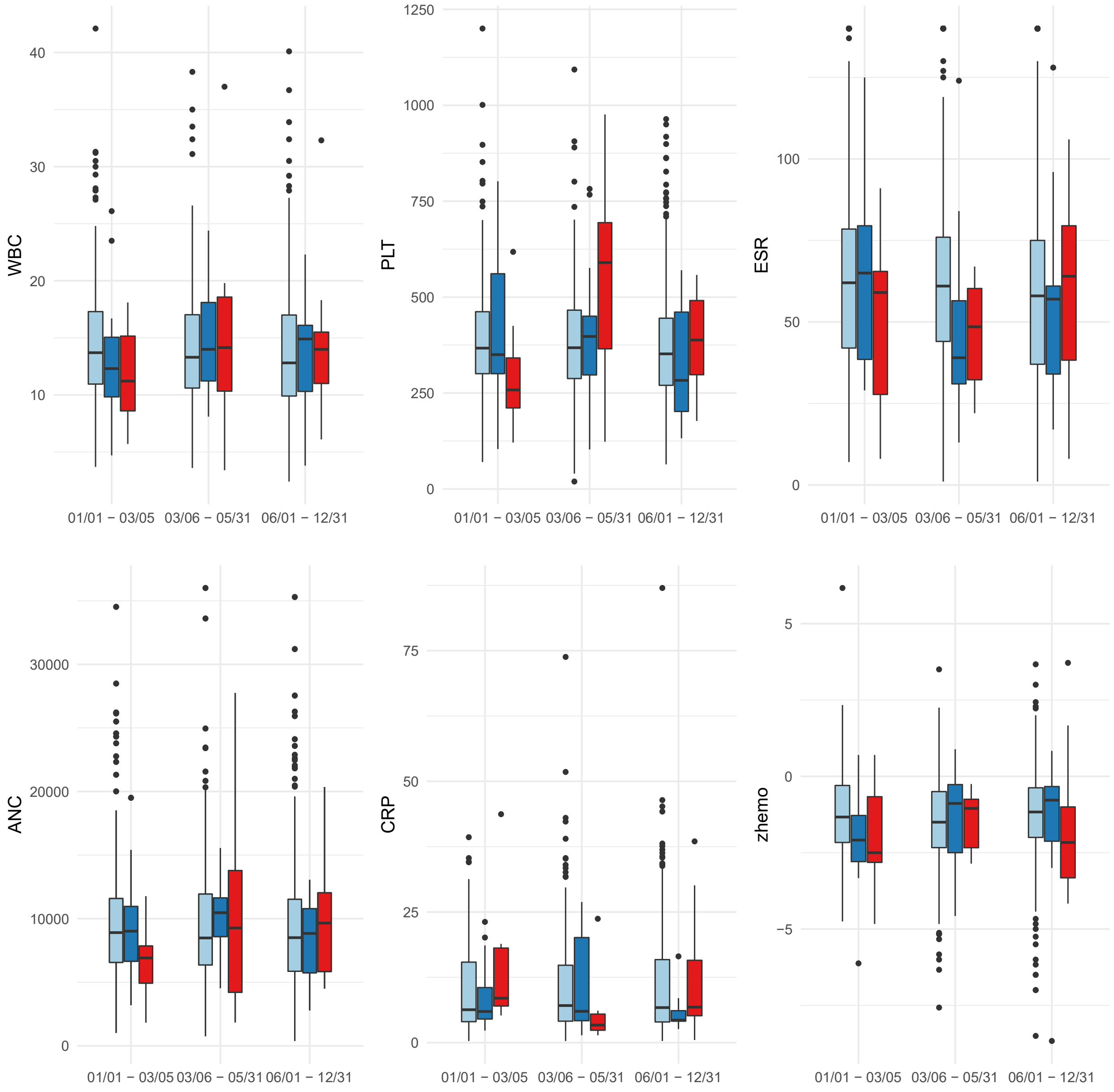

Comparison of laboratory values for KD patients in the 2002-2019 baseline period (light blue boxes), 2020 (dark blue boxes), and 2021 (red boxes). Black horizontal line is median, box is IQR, and whiskers are the 95% with outliers shown as black dots. (Numbers of patients for each time period are shown in eTable 2.) There were no statistically significant differences across years by time period (Kruskal-Wallis tests). Abbreviations: WBC: white blood cell count, ESR: erythrocyte sedimentation rate, ANC: absolute neutrophil count, CRP: C-reactive protein, Zhemo: hemoglobin Z-score.

© 2022 Burney JA et al. *JAMA Network Open.*

eFigure 10. Clinical Presentation of Patients With KD in 2020 and 2021, Compared With 2002-2019 Baseline, by Pandemic-Defined Season

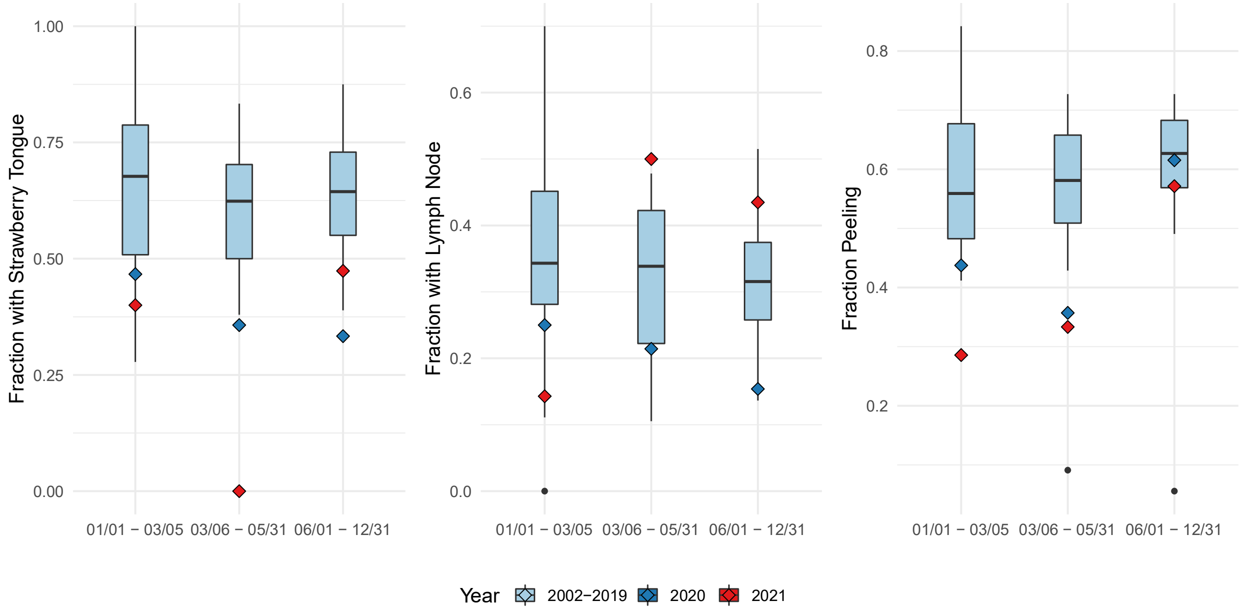

Characteristics of clinical presentation of KD patients by pandemic-defined periods, historical period, (light blue boxes, median and IQR of 18 years), 2020 (dark blue diamond), and 2021 (red diamond). Numbers of patients are provided in eTable 3.

© 2022 Burney JA et al. *JAMA Network Open.*

eFigure 11. Geographic Distribution of Patients With KD in RCHSD Data Set, 2019-2021

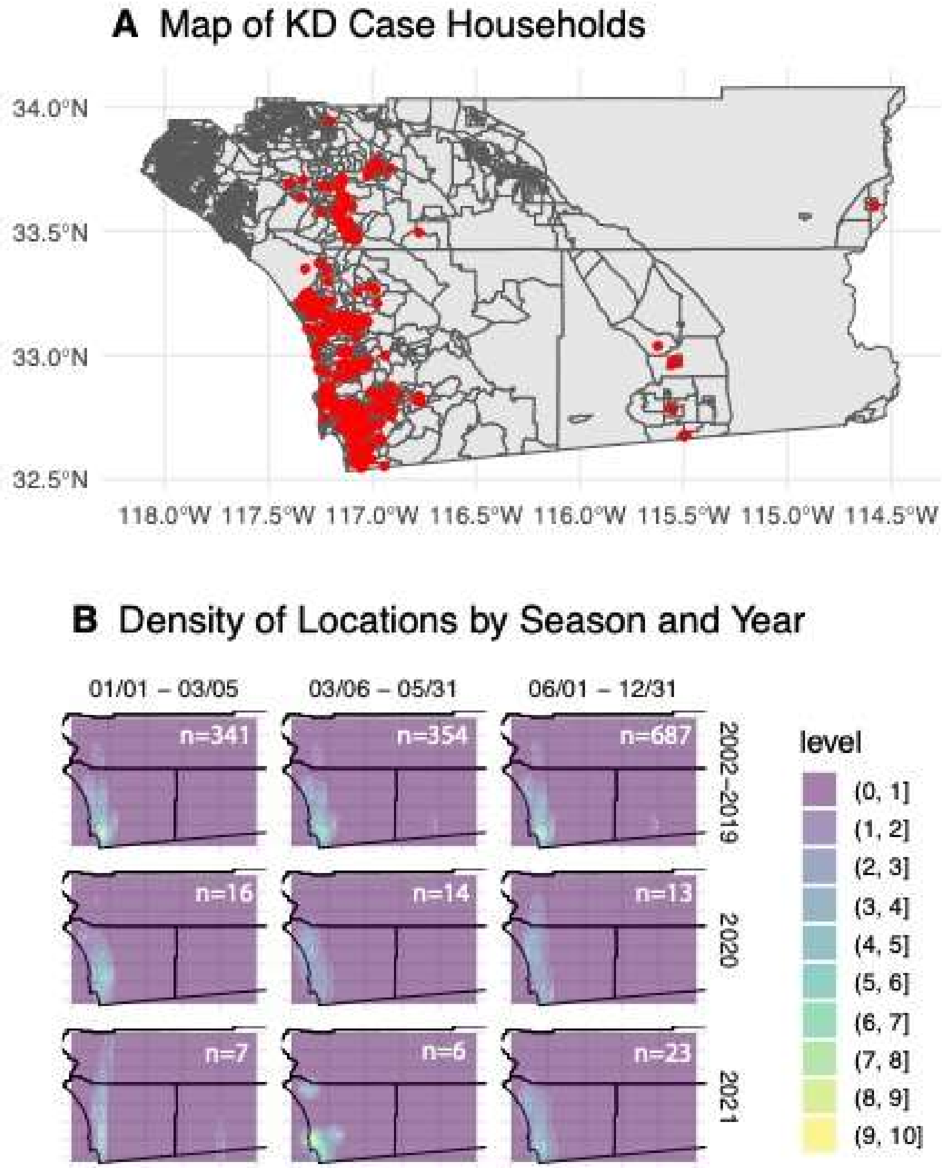

A: Map of 4744 Census Block Groups across the three southernmost California counties (San Diego, Imperial, Riverside). Dark grey outlines show CBG borders. Red dots show residences of all KD cases between 2019-2021. B: Normalized geographic distributions of KD case residences by pandemic periods defined in 2020 and year. Some of the shift northwards during the pandemic reflects a longer-run trend in populations in San Diego County, but the distribution shifts back towards historical bounds in spring-summer 2021.

© 2022 Burney JA et al. *JAMA Network Open.*

eFigure 12. Association Between Census Block Group–Level Median Household Income and Median Time Spent Away From Home

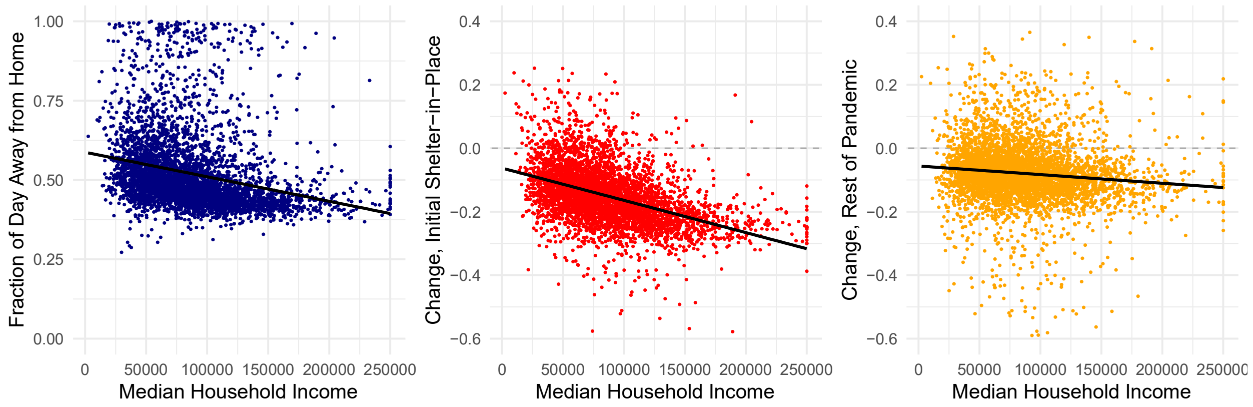

The relationship between income and time spent away from home in Southern California (4744 CBGs) over the pre-pandemic period in 2020 (left, blue) and different phases of the pandemic in 2020 (red=shelter-in-place, yellow=remainder of 2020). The relationship is highly significant (p<1e-14 for each) but the slope for the latter part of 2020 is ~29% of the slope for the initial shelter-in-place period (-2.9e-7 vs. -1.0e-6).

© 2022 Burney JA et al. *JAMA Network Open.*

eFigure 13. KD Fever Onset by Day of Week

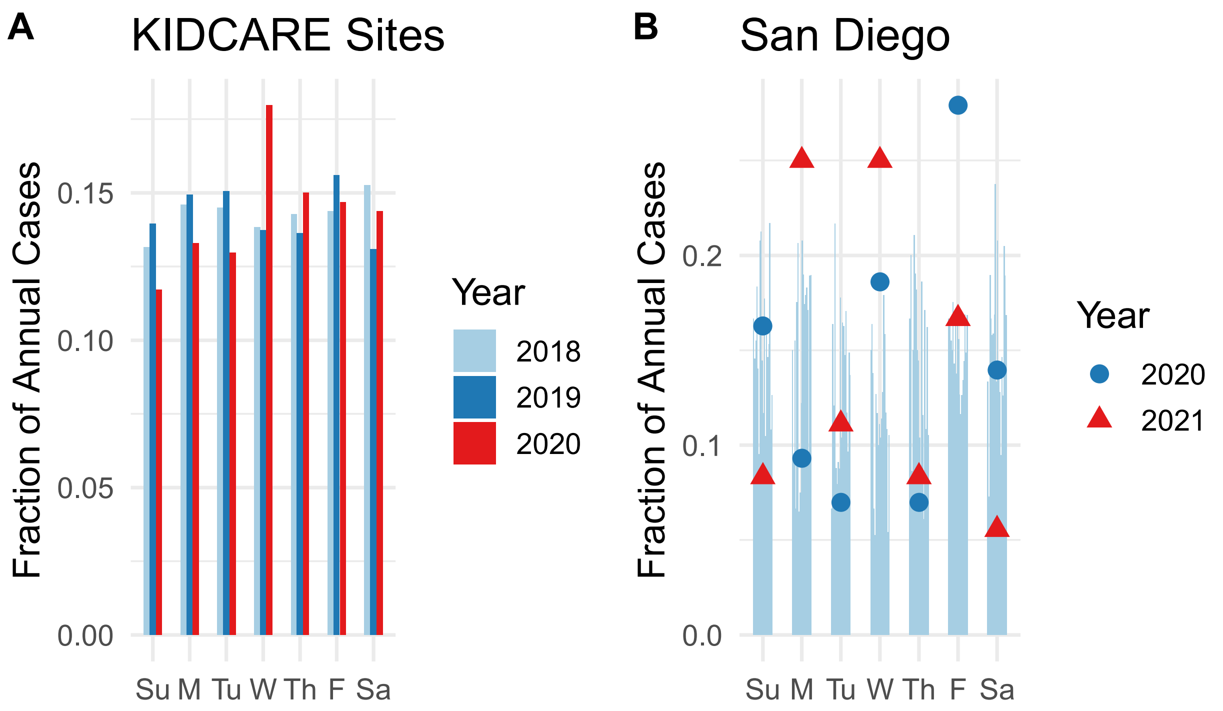

KD onset of fever by day of week, historically and during pandemic period, for KIDCARE sites and San Diego. For San Diego, light blue bars show each year 2002-2019. Due to the small number of years for the multi-site study (A) and the small numbers of cases during 2020 and 2021 in the RCHSD dataset (B) we do not assess statistical significance.

© 2022 Burney JA et al. *JAMA Network Open.*
