# Supplementary material for: Epidemiological and Clinical Features of Kawasaki Disease During the COVID-19 Pandemic in the United States
Source: JAMA Netw Open. 2022 Jun 17;5(6):e2217436. doi: 10.1001/jamanetworkopen.2022.17436 (PMC9206189; doi:10.1001/jamanetworkopen.2022.17436)
Supplement: Supplement 2. — Nonauthor Collaborators [file jamanetwopen-e2217436-s002.pdf]

Supplemental Online Content: Nonauthor Collaborators
*First name, last name, and suffix (if applicable) are required and will appear in PubMed.

***Group Name(s): KIDCARE Study Investigators**

***First Name and Middle Initial(s)**	***Last Name**	***Suffix (eg, Jr, III)**	Academic Degrees	Institution	Location (city, state/province, country)	Role or Contribution, eg, chair, principal investigator	Group (if more than 1 Group listed in the byline) and/or Subgroup (eg, Steering Committee)
Moshe	Arditi		MD	Cedars-Sinai Medical Center, Los Angeles, CA	Los Angeles, CA	Co-investigator	
Jackie	Szmuszkovicz		MD	Children's Hospital Los Angeles, CA	Los Angeles, CA	Co-investigator	
Negar	Ashouri		MD	Children's Hospital Orange County, CA	Orange, CA	Co-investigator	
Sylvia	Yeh		MD	Harbor UCLA, CA	Torrance, CA	Co-investigator	
David	Michalik		MD	Miller Children's Hospital Long Beach, CA	Long Beach, CA	Co-investigator	
Jane	Burns		MD	Rady Children's Hospital San Diego, CA	San Diego, CA	Co-investigator	
Adriana	Tremoulet		MD	Rady Children's Hospital San Diego, CA	San Diego, CA	Co-investigator	
Michael	Portman		MD	Seattle Children's, WA	Seattle, WA	Co-investigator	
Charles	Newcomer		MD	Mattel Children's Hospital, UCLA, CA	Los Angeles, CA	Co-investigator	
Gregory	Kurio		MD	Benioff Children's Hospital, UCSF-Oakland, CA	Oakland, CA	Co-investigator	
Amy	McNellis		MD	Benioff Children's Hospital, UCSF-San Francisco, CA	San Francisco, CA	Co-investigator	
Katherine	Kim		MD	University of California Davis, CA	Davis, CA	Co-investigator	
Jocelyn	Ang		MD	Children's Hospital of Michigan, MI	Detroit, MI	Co-investigator	
John	Manaloor		MD	Indiana University,IN	Bloomington, IN	Co-investigator	
Anne	Rowley		MD	Lurie Children's Hospital, Chicago, IL	Chicago, IL	Co-investigator	
Guliz	Erdem		MD	Nationwide Children's Hospital, Columbus, OH	Columbus, OH	Co-investigator	
Allison	Bartlett		MD	University of Chicago, Chicago, IL	Chicago, IL	Co-investigator	
Madan	Kumar		MD	University of Chicago, Chicago, IL	Chicago, IL	Co-investigator	
Jane	Newburger		MD	Boston Children's Hospital, MA	Boston, MA	Co-investigator	
Rana	El Feghaly		MD	Boston Children's Hospital, MA	Boston, MA	Co-investigator	
Emily	Ansusinha		MD	Children's National Hospital, Washington DC	Washington DC	Co-investigator	
Roberta	DeBiasi		MD	Children's National Hospital, Washington DC	Washington DC	Co-investigator	
Ashraf S.	Harahsheh		MD	Children's National Hospital, Washington DC	Washington DC	Co-investigator	
Tova	Ronis		MD	Children's National Hospital, Washington DC	Washington DC	Co-investigator	
Supriya	Jain		MD	Maria Fareri Children's, NY	Valhalla, NY	Co-investigator	
Marsha	Anderson		MD	Children's Hospital Colorado, CO	Aurora, CO	Co-investigator	
Samuel	Dominguez		MD	Children's Hospital Colorado, CO	Aurora, CO	Co-investigator	
Michelle	Hite		MD	Children's Hospital Colorado, CO	Aurora, CO	Co-investigator	

Supplemental Online Content: Nonauthor Collaborators
*First name, last name, and suffix (if applicable) are required and will appear in PubMed.

***First Name and Middle Initial(s)**	***Last Name**	***Suffix (eg, Jr, III)**	Academic Degrees	Institution	Location (city, state/province, country)	Role or Contribution, eg, chair, principal investigator	Group (if more than 1 Group listed in the byline) and/or Subgroup (eg, Steering Committee)
Pei-Ni	Jone		MD	Children's Hospital Colorado, CO	Aurora, CO	Co-investigator	
Kari	Simonsen		MD	University of Nebraska Medical Center, NE	Omaha, NE	Co-investigator	
Archana	Chatterjee		MD	University of South Dakota-Sanford, SD	Vermillion, SD	Co-investigator	
Dongngan	Truong		MD	University of Utah, UT	Salt Lake City, UT	Co-investigator	
Jose	Romero		MD	Arkansas Children's Hospital, AR	Little Rock, AR	Co-investigator	
David	Lloyd		MD	Emory University, Atlanta, GA	Atlanta, GA	Co-investigator	
Nichole	Samuy		MD	University of Alabama, AL	Tuscaloosa, AL	Co-investigator	
Paul	Scalici		MD	University of Alabama, AL	Tuscaloosa, AL	Co-investigator	
Kavita	Sharma		MD	University of Texas Southwestern, Dallas, TX	Dallas, TX	Co-investigator	
Natasha	Halasa		MD	Vanderbilt, Nashville, TN	Nashville, TN	Co-investigator	
